# The effect of traditional Thai massage vs routine physical therapy on gait pattern in spastic cerebral palsy: A cross-over randomized controlled trial

**DOI:** 10.1371/journal.pone.0325169

**Published:** 2025-05-29

**Authors:** Peerapat Lertwiram, Chanika Angsanuntsukh, Krongkaew Supapitanon, Tanyaporn Patathong, Apiphan Iamchaimongkol, Suchanont Baosuwan, Ponsaphat Ongtanasin, Phimpisut Srinorasit, Patarawan Woratanarat

**Affiliations:** 1 Department of Orthopaedics, Faculty of Medicine Ramathibodi Hospital, Mahidol University, Bangkok, Thailand; 2 Department of Rehabilitation Medicine, Faculty of Medicine Ramathibodi Hospital, Mahidol University, Bangkok, Thailand; 3 Complementary and Alternative Medicine Unit, Outpatient Department, Faculty of Medicine Ramathibodi Hospital, Mahidol University, Bangkok, Thailand; Prince Sattam bin Abdulaziz University, SAUDI ARABIA

## Abstract

**Background:**

Various types of massage, including the traditional Thai, have not yet provided conclusive evidence to reduce the spasticity and improve walking ability for cerebral palsy (CP).

**Objectives:**

To assess the effect of traditional Thai massage (TM) vs. a standard physical therapy (PT) on gait pattern in spastic CP.

**Methods:**

A cross-over single blinded randomized controlled trial was conducted between October 2022 and October 2023 (Thai Clinical Trials Registry: TCTR20220530007). Individuals with aged 5 years or older, and diagnosed as spastic CP, Gross Motor Function Classification System (GMFCS) I-III were recruited. Participants were randomly assigned into group A (TM followed by PT), and group B (PT followed by TM). Each treatment lasted for 6 weeks (Phase I-II), with a 6-week washout period. Lower extremity range of motion, muscle tone, electromyography, gait profile score (GPS), oxygen consumption was blindly assessed and intention-to-treat analyzed.

**Results:**

From 32 eligible patients (16 cases per group), average age was 16.1 ± 9.8 years (group A), and 13.6 ± 5.8 years (group B). Group B demonstrated higher left ankle dorsiflexion than group A (1.7 ± 13.0 degrees vs 12.1 ± 6.9 degrees, *P*-value = 0.011). GPS slightly improved in Phase I, and contradictory enhanced in TM while deteriorated in PT in Phase II without significant differences between groups. After adjusted for ankle dorsiflexion, TM significantly provided less overall GPS (-1.6 (95% confidence interval (CI) -2.6, -0.6), *P*-value = 0.001), and higher peak activity of right rectus femoris (0.132 mV (95%CI 0.001, 0.262), *P*-value = 0.030) compared to PT. Other outcomes did not differ significantly between two treatments, and no complication was detected.

**Conclusions:**

TM has a positive effect on gait performance, as indicated by GPS and increased activity in the right rectus femoris muscle when compared to PT. A large-scale non-inferiority trial is required to affirm the impact of TM.

## Introduction

In developing countries, the prevalence of cerebral palsy (CP) was up to 3.34% [1]. As non-progressive insults to an immature brain, cerebral palsy encompasses posture and movement abnormalities. Common antenatal and perinatal causes arise from maternal high blood pressure, infections, toxin, metabolic disorders, prolonged labor, abnormal presentation, and low birth weight [2,3]. The nervous and muscular systems are affected in various forms, i.e., spastic, hypotonic, dyskinetic, rigidity, ataxic, and mixed types. Understanding types of CP, particularly for spasticity is crucial for appropriate treatment strategies [4], mainly intensive physical therapy (PT) with additional medication, bracing, and surgery.

PT and complementary massage may alleviate pain, stiffness, and improve ambulatory function. The former usually combines muscle stretching and strengthening, while the latter may involve stretching, compression, kneading, tapping, or vibration. Prolonged passive muscle stretching could stimulate Golgi tendon, and enhance flexibility of soft tissues and joint motion by omitting alpha motor neuron function [5–7]. Muscle strengthening can build muscle power by progressive resistant actions [8]. Deep cross friction method induced hyperemia, and improved pain and sensation [9], whereas gentle massage facilitated muscle lengthening and voluntary contraction [10]. Besides, mechanically stimulated oscillatory motions by body vibration was transmitted to muscle fibers, and then inhibited alpha neurons to reduce spasticity [7].

Therapeutic effects of several PT techniques have been widely explored. From previous systematic reviews [11–13], PT with lower extremity/core body therapy, or gait training/treadmill reinforced balance, coordination [13], gross motor function [12], and gait [11–13] in children with CP. Also, resistance exercise for strengthening significantly increased GMFM when compared to controls/conventional therapy (mean difference; MD 1.73 (95% confidence interval; CI 0.81, 2.64), *P*-value < 0.001) [14]. Based on the review of 34 systematic reviews, lower-limb directed/functional, strengthening, and gait training were suggested to improve gait speed in CP [15].

Not only PT, but also massage for children with CP has been introduced for many years [16]. Various techniques such as deep cross friction, Swedish, and Pakistani massage, showed contradictory results of the spasticity (the modified Ashworth scale; MAS), abnormal reflex, or Gross Motor Function Measure 88 (GMFM-88) when compared to PT [5,9,17–19]. A systematic review [20] reported muscle tone reduction at arm, hip adductors, and quadriceps femoris as well as increased GMFM after massage in children with CP. When compared to PT, whole-body stroking [18] and whole-body Pakistani massage significantly reduced MAS [19] while whole-body Swedish [21] and deep cross-friction lower limb massage [9] did not reach significant difference. Additionally, lower-limb Pakistani massage noteworthy improved GMFM while whole-body Pakistani technique was not superior to PT. Combined passive stretching with whole body vibration for 6 weeks tended to improved MAS, muscle strength and balance when compared to passive stretching alone [7].

Traditional Thai massage (TM) is an alternative treatment option. Gentle compression and stretching along muscle mass (neck, back, upper and lower extremities) can alleviate stress and anxiety by stimulating the parasympathetic nervous system [5]. Incorporating TM could relief muscle rigidity, enhance walking abilities. In stroke patients, it can effectively reduce muscle rigidity, enabling affected limbs usage, and enhancing overall quality of life [22]. Applying TM in a CP-case series significantly improved MAS from the baseline status [5]. A randomized controlled trial reported quadriceps MAS reduction without walking difference when compared 30-minute TM to static muscle stretching of both legs [23]. Nevertheless, the previous studies [5,23] had need of proper randomization process, concealment, blinding of outcome assessors, adequate study duration, and sample size.

Regarding limited evidences of TM vs PT, this study was aimed to compare the effects of TM and conventional PT on improving gait in individuals with spastic CP. A Gait Profile Score (GPS) derived from 9 Gait Variable Scores (GVS) assessed by three-dimensional (3D) gait analysis. MAS, temporal spatial data, kinetics, oxygen consumption, electromyography, and pedobarography were considered. Aforementioned massage, TM was hypothesized to enhance walking abilities arising from muscle spasticity by lowering GPS, MAS, oxygen consumption; increasing gait velocity; and normalizing joint motions (kinematics), muscle contraction, and foot pressures when compared to PT. This strategy may provide greater access to care, and ease the burden on PT teams, especially in the rural areas [24].

## Materials and methods

### Patient selection and study design

A cross-over designed single blinded randomized controlled trial (RCT) ratio 1:1 was conducted at the university hospital between 1 October 2022 and 31 October 2023 (S1 and S2 Protocol). Patients with spastic CP aged five years or older and classified as Gross Motor Function Classification System (GMFCS) levels I to III were recruited. Patients with body temperature more than 38 degrees Celsius, bleeding tendencies, unhealed fractures, contact dermatitis, skin infection, fixed joint contracture, a history of botulinum toxin injection within last 6 months, attention deficit hyperactive disorder, behavioral disorder, and uncontrolled seizure were excluded. All participants were provided written informed consents or assent forms and consents from parents’ or guardians where appropriate. The study received full approval from the Institutional Review Board (COA. MURA2021/753), and was prospectively registered at Thai Clinical Trials Registry (TCTR20220530007).

After generating a various-block randomization by STATA 16.1 (PW) and placing the allocations in sequentially numbered sealed opaque envelopes, patients were recruited (PL) and randomly assigned to either group A (TM-PT) or group B (PT-TM). Each intervention took 6 weeks, with a 6-week gap time between intervention phases. During the washout period, the administration of any additional treatment apart from the given home program was not allowed. In cases of inconvenient outpatient department (OPD) visits, guardians were provided a treatment protocol according to their randomization group. The protocol video instructed details exactly the same as the treatment provided in the OPD. The instruction brochure and the demonstration video according to each treatment time period were provided to home-based participants. Weekly phone calls and telemedicine were also used to ensure proper treatment program and encounter any possible problem.

### Traditional Thai massage protocol

Subjects underwent the 30-minute TM twice a week for a total duration of 6 weeks. The developed protocol (Table 1) was based on the Court-type traditional Thai massage (CTTM) which included basic massage lines, open wind gait, and major signal points along neck, back, and all extremities. Two 3-year experienced traditional Thai medicine practitioners evaluated physical conditions and emotional states of the participants before commencing treatment. The massage using fingertips and the base of the palm pressured on the targeted areas. The compression was adjusted in accordance with the spasticity and the individual’s age. Gentle muscle stretching was also applied. The aim was to ensure the recipient of the therapy felt maximally comfortable and experienced no sensations of crushing, twisting, or excessive pressure on the muscles.

**Table 1:** A 30-minute Traditional Thai massage protocol involving muscles of upper-lower limbs, cervical (C) and lumbar (L) levels.

Step	Position	Massage details			Duration (minutes)
		Basic line (10 seconds/area)	Major signal point (30 seconds/point)	Open wind gait	
1	Supine	Leg – thigh - leg	Anterior ankle	Both groins	5
2	Lateral decubitus	Paravertebral muscles (L5 – C7)	–	–	5
3	Lateral decubitus	–	Lateral thigh, leg	–	5
4	Lateral decubitus	–	Medial thigh, leg	–	5
5	Supine	Lateral arm - forearm	–	–	2
6	Supine	Medial arm - wrist	–	–	2
7	Sitting	Shoulder – neck (C7)	–	–	2
8	Sitting	C7 - occiput	–		1
9	Sitting	–	Nape of neck, occiput, forehead	–	2
10	Sitting	–	Upper chin, philtrum	–	1
Total	3	6	5	1	30

### Physical therapy protocol

Physical therapy was conducted by a 5-year experienced physical therapist. The 40-minute intervention applied for 3 sessions per week for a total 6 weeks. Each session consisted of 10 minutes of stretching, 15 minutes of strengthening, and 15 minutes of ambulatory training. Specific muscle groups were stretched and strengthen based on individual needs, i.e., the gastrosoleus complex, tibialis posterior, peroneal muscles, rectus femoris, hamstring, hip adductor muscles, iliopsoas muscle, and biceps brachii muscles.

### Home program

Participants were instructed to perform physical therapy at home (30-minutes session, five times (150 minutes) per week). Stretching exercises targeted both upper and lower extremities for 30 seconds, repeated 3 times per muscle group. For strengthening exercises, patients were directed to perform shoulder abduction/flexion, elbow flexion/extension, hip extension/abduction, and knee extension/flexion. Each movement was repeated multiple times for 3–5 minutes per joint on both sides. Ambulatory training involved practicing arm and leg movements, forward, backward, left, and right walking motions. The use of walking aids was permitted if available.

### Data collection

Baseline characteristics including age, sex, weight, height, spastic CP classification (diplegia, hemiplegia, and quadriplegia), GMFCS level, birth weight, delivery method, birth hospital, and any perinatal complications were retrieved. In regards with pre-, and post-therapeutic session at 0, 6, 12, and 18 weeks, clinical examination, baseline range of motion (ROM), muscle tone by MAS, gait kinematics, temporal-spatial parameters, GPS, surface electromyography (sEMG), dynamic, maximum rate of oxygen consumption (VO_2_max) measurements, and static foot pressure were assessed by 2 physiotherapists who were blinded to the intervention process.

Participants were equipped with 29 reflective markers according to the modified Helen Hayes [25]. They were instructed to walk approximately 8 meters in a straight line for 10–15 rounds. Joint motion and gait parameters were captured by 8 cameras (3D motion capture, Cortex 6.2 software). Three best gait cycles were selected, and analyzed using OrthoTRack 6.61 (Motion Analysis Corporation, Santa Rosa, CA, USA). Bipolar Ag/AgCl surface electrodes (3M Red dot, size 35 × 40 cm) were applied on hip, rectus femoris, hamstring, anterior tibialis, and posterior gastrocnemius muscles. Electromyography of each muscle group was recorded during maximum muscle voluntary contraction and walking (ProEMG software and Myon 320 wireless EMG, Myon AG, Schwarzenberg Switzerland). Oxygen consumption was measured as VO_2_max (ml/min/kg) using the Oxycon mobile apparatus, CareFusion, Germany. Foot pressure during standing and walking on the pedobarograph, Sensor Medica (Rome, Italy) was collected.

GPS as the primary outcome was estimated based on kinematics data (pelvic tilt, pelvic rotation, pelvic obliquity, hip flexion/extension, hip abduction/adduction, hip rotation, knee flexion/extension, ankle dorsiflexion/plantar flexion, and foot progression).The GPS of both sides (right/left), and overall gait profiles were computed from the root mean square differences against the non-pathological gait reference data set [26]. Participants’ compliance, and complications (soft tissue injury, ecchymosis, muscle soreness, pain, inflammation, stiffness, joint swelling [22,27]) were documented and monitored through phone calls, and a logbook administered by parents. Other co-interventions were informed and encourage all participants to be avoided.

### Statistical analysis

Patient characteristics and other outcomes were summarized using mean with standard deviation for continuous variables, and frequencies with percentages for categorical variables. In the baseline data, continuous variables were compared between groups using paired t-tests, while categorical variables were assessed using either Chi-square or Fisher’s exact tests. According to the repeated measurement analysis, a multilevel mixed-effects linear regression model was employed for continuous data and a multilevel mixed-effects Poisson regression model was utilized for analyzing categorical variables. The primary analysis was based on the principle of intention-to-treat. Imbalance baseline characteristics, prognostic factors, and possible confounders were controlled in multilevel mixed-effects models. Adjusted mean, standard error (SE), mean difference (MD) and 95% confidence interval (CI) were reported. All analyses were performed using STATA 18.0, Statacorp, College Station, Texas, USA. *P*-value of less than 0.05 was considered as statistical significance.

### Sample size calculation

The sample size of 26 cases was calculated based on alpha error 0.05, beta error 0.2, GPS mean ± standard deviation (SD) of PT group 13.90 ± 4.9 [28], and TM group 11.12 ± 1.3. Total 32 cases were accounted for a 20% anticipated loss to follow-up. The mean GPS difference between the PT and TM groups for the alternative hypothesis at 2.78 with Minimally Clinically Important Difference (MCID) of 1.7.[26,28,29].

## Results

**Fig 1:**
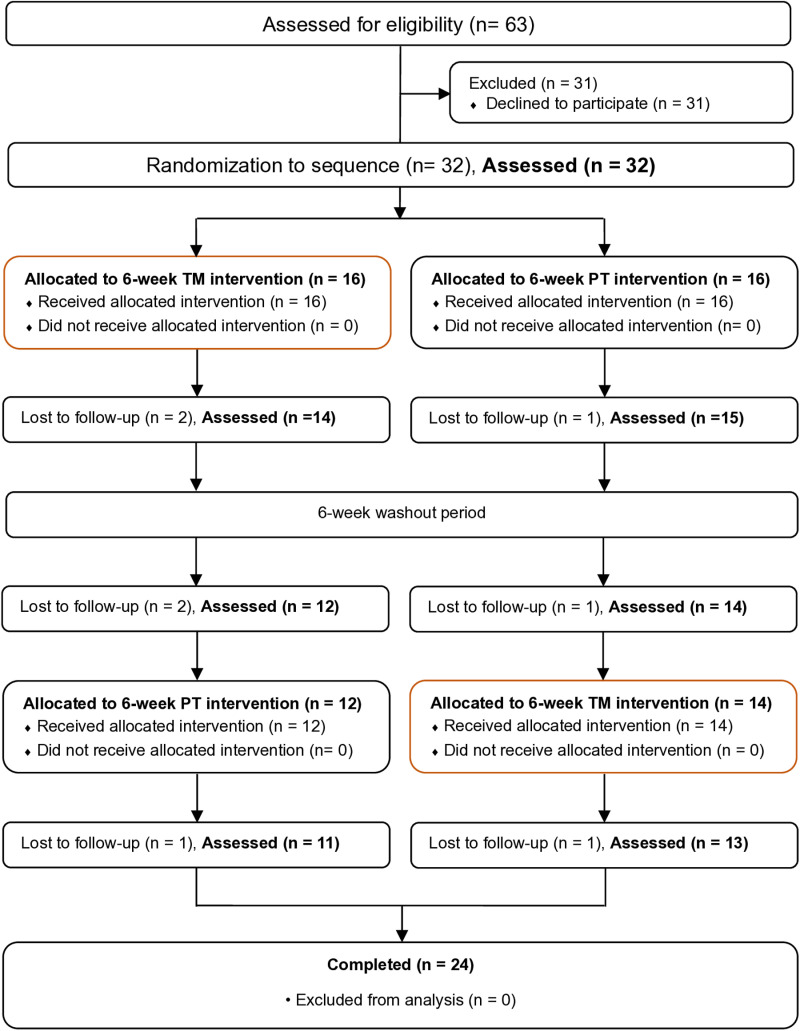
CONSORT diagram.

From 63 eligible patients, 32 patients were willing to participate, and 31 of them refused. Average age (32 cases, 14.8 ± 8.2 years vs. 31 cases, 14.9 ± 5.7 years, *P*-value = 0.9867) and male distribution (21/32 cases (65.6%) vs. 19/31 cases (61.3%), *P*-value = 0.7209) were comparable between respondents and non-respondents. Thirty-two patients were randomized with 16 patients allocated in group A (TM-PT) and 16 patients in group B (PT-TM). At 6, 12, and 18 weeks, 3, 3, and 2 patients were subsequently lost to follow up, respectively. At the end, the follow up rate was 68.8% in group A, and 81.3% in group B. Enrollment, treatment, sequence, allocation and follow-up were summarized as a CONSORT diagram [30] (Fig 1), and CONSORT checklist (S1 CONSORT Checklist).

The mean age was 16.1 ± 9.8 years for group A, and 13.6 ± 5.8 years for group B (Table 2). In regards with group A and group B, most of them were male (68.8% vs 62.5%), diplegia (50.0% vs 56.3%) and categorized in GMFCS II (56.2% vs 43.8%). Birth weight of group A (2937.5 ± 928.7 g) was lower than that of group B (3437.5 ± 813.9 g), *P*-value = 0.116. Twenty-five percent of both groups used gait aid, and video home program was applied in 11 (68.8%) patients in group A and 12 (75.0%) patients in group B. No significant differences of baseline characteristics including GMFCS, birth weight, perinatal complications, gait aid, video home program between randomization groups.

**Table 2:** Baseline patient characteristics for the two groups.

Variable	Group A (TM-PT) N = 16	Group B (PT-TM) N = 16	*P*-value
Age (year), mean (SD)	16.1 (9.8)	13.6 (5.8)	0.850
Male (%)	11 (68.8)	10 (62.5)	0.710
Weight (kg), mean (SD)	37.9 (16.3)	36.2 (10.6)	0.738
Height (cm), mean (SD)	143.9 (18.0)	143.4 (18.7)	0.934
Classification (%)			1.000
- Diplegia	8 (50.0)	9 (56.3)	
- Hemiplegia	6 (37.5)	5 (31.3)	
- Quadriplegia	2 (12.5)	2 (12.5)	
GMFCS (%)			0.498
- I	3 (18.8)	6 (37.5)	
- II	9 (56.2)	7 (43.8)	
- III	4 (25.0)	3 (18.7)	
Birth weight (g), mean (SD)	2937.5 (928.7)	3437.5 (813.9)	0.116
Delivery mode (%)			0.723
- Normal labor	8 (50.0)	7 (43.8)	
- Cesarean section	8 (50.0)	9 (56.3)	
Birth at public hospital (%)	11 (68.8)	10 (62.5)	0.710
Perinatal complication (%)	9 (56.3)	7 (43.8)	0.480
Gait aid (%)	4 (25.0)	4 (25.0)	1.000
Video home program (%)	11 (68.8)	12 (75.0)	1.000

TM = Thai massage, PT = physiotherapy, SD = standard deviation, GMFCS: Gross Motor Function Classification System

Group A were significantly limited left ankle dorsiflexion than group B at knee extension (1.7 ± 13.0 degrees vs 12.1 ± 6.9 degrees, *P*-value = 0.011) and at knee flexion (8.4 ± 11.9 degrees vs 18.8 ± 10.4 degrees, *P*-value = 0.014), Table 3. Other baseline characteristics, clinical examination, motions, gait parameters, muscle tone, sEMG, GPS, foot pressure, and VO_2_max of both groups demonstrated similar patterns (S1-S4 Tables in S1 File).

**Table 3:** Baseline range of motion comparing between randomized groups.

ROM (degrees), mean (SD)	Right side			Left side		
	Group A (TM-PT) N = 16	Group B (PT-TM) N = 16	*P*-value	Group A (TM-PT) N = 16	Group B (PT-TM) N = 16	*P*-value
Hip flexion	124.7 (14.9)	126.3 (9.9)	0.729	124.7 (15.9)	125.9 (8.6)	0.784
Hip extension	21.3 (6.5)	23.4 (5.7)	0.317	22.8 (6.6)	23.8 (18.3)	0.582
Hip abduction	39.4 (9.8)	37.8 (8.0)	0.624	37.2 (11.8)	38.1 (8.3)	0.797
Hip adduction	24.7 (5.6)	26.6 (7.2)	0.420	27.2 (12.8)	24.7 (6.7)	0.495
Hip internal rotation	65.3 (14.9)	62.5 (11.8)	0.559	61.9 (15.0)	63.8 (10.1)	0.682
Hip external rotation	54.7 (16.3)	60 (13.9)	0.329	60.9 (29.0)	62.2 (14.1)	0.878
Knee flexion	139.4 (7.0)	140.9 (6.4)	0.516	139.1 (7.1)	140 (8.2)	0.732
Knee extension	-1.4 (6.9)	-1.8 (6.7)	0.665	-0.5 (6.3)	-1.0 (5.0)	0.422
Ankle dorsiflexion at KE	3.6 (17.8)	12.1 (7.7)	0.374	1.7 (13.0)	12.1 (6.9)	0.011*
Ankle dorsiflexion at KF	10.3 (11.6)	18.1 (10.9)	0.060	8.4 (11.9)	18.8 (10.4)	0.014*
Ankle plantar flexion	67.2 (10.9)	68.1 (9.5)	0.797	70.0 (12.1)	70.6 (11.8)	0.884
Ankle inversion	51.9 (15.7)	52.2 (8.9)	0.945	51.3 (15.8)	50.0 (12.1)	0.803
Ankle eversion	40.6 (8.7)	40 (14.3)	0.882	41.9 (12.2)	37.5 (10.0)	0.277
Popliteal angle	43.8 (11.0)	42.5 (18.5)	0.818	43.1 (12.1)	40.6 (15.3)	0.611

TM = Thai massage, PT = physiotherapy, KE = knee extension, KF = knee flexion *significant *P*-value < 0.05

**Fig 2:**
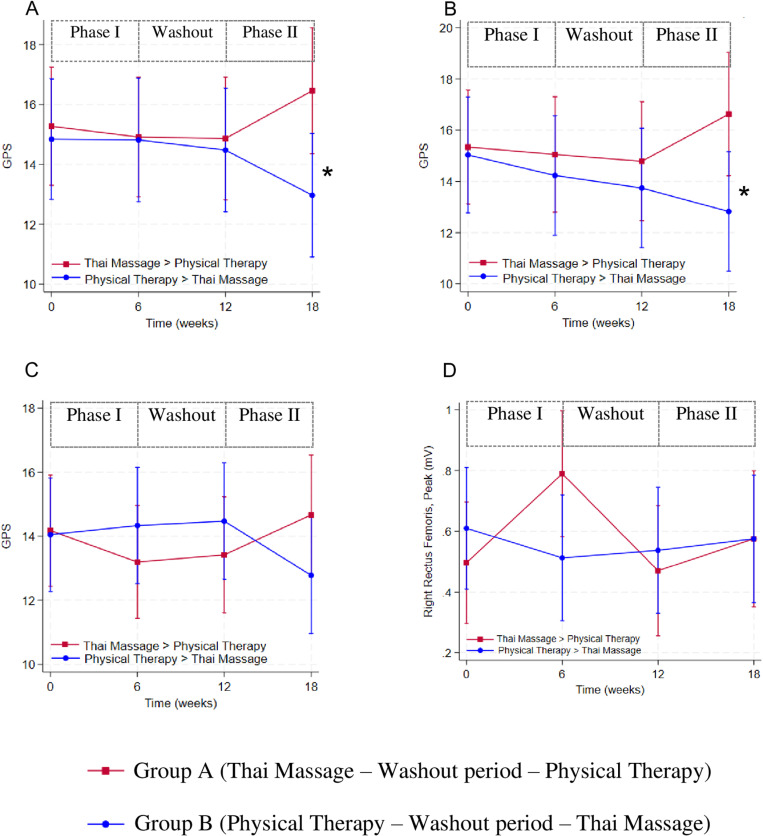
Gait Profile Score (GPS) during 18 weeks, (A) overall GPS, (B) right GPS, (C) left GPS, and (D) peak activity of right rectus femoris muscle from surface electromyograph (sEMG), expressed as mean (dot), standard error; SE (line), and significant *P*-value < 0.05 (*).

Mixed-effects regression adjusted by randomization groups and visits were applied on the basis of the analysis. At the end of follow-up (the final visit), overall GPS (MD -3.4 (95%CI -6.3, -0.5), *P*-value = 0.023), and right GPS (MD -3.8 (95%CI -7.1, -0.5), *P*-value = 0.025) significantly reduced in group B (PT-TM) when compared to group A (TM-PT), Fig 2. However, Phase I and II showed contradictory results. In Phase I, GPS of both treatments seemed to be slightly improved. On the opposite way in Phase II, GPS was enhanced in TM while it was deteriorated in PT. After taken all visits of both phases into account, overall and right GPS did not differ between groups (overall GPS MD = -0.4 (95%CI -3.3, 2.4), *P*-value = 0.764, right GPS MD = -0.3 (95%CI -3.5, 2.9), *P*-value = 0.850). Besides, the post-hoc Bonferroni test for multiple comparisons of GPS and right rectus femoris peak activity at each point of time of both phases also insignificantly differ between randomization groups, including TM vs PT at the second visit, and PT vs TM at the final visit (S5 Table in S1 File).

According to the cross-over design, both GPS and peak activity of the right rectus femoris muscle reverted to their baseline levels at the 6-week washout period indicated no carry over effects. The effects of provided home program during the washout period (6–12 weeks) did not differ significantly between treatment groups at 6 weeks (GPS -0.084 (95%CI -3.025, 2.857), *P*-value = 0.955, peak activity of the right rectus femoris muscle -0.277 (95%CI -0.571, 0.017), *P*-value = 0.065) and at 12 weeks (GPS -0.385 (95%CI -3.360, 2.590), *P*-value = 0.800, peak activity of the right rectus femoris muscle 0.067 (95%CI -0.233, 0.366), *P*-value = 0.662).

**Fig 3:**
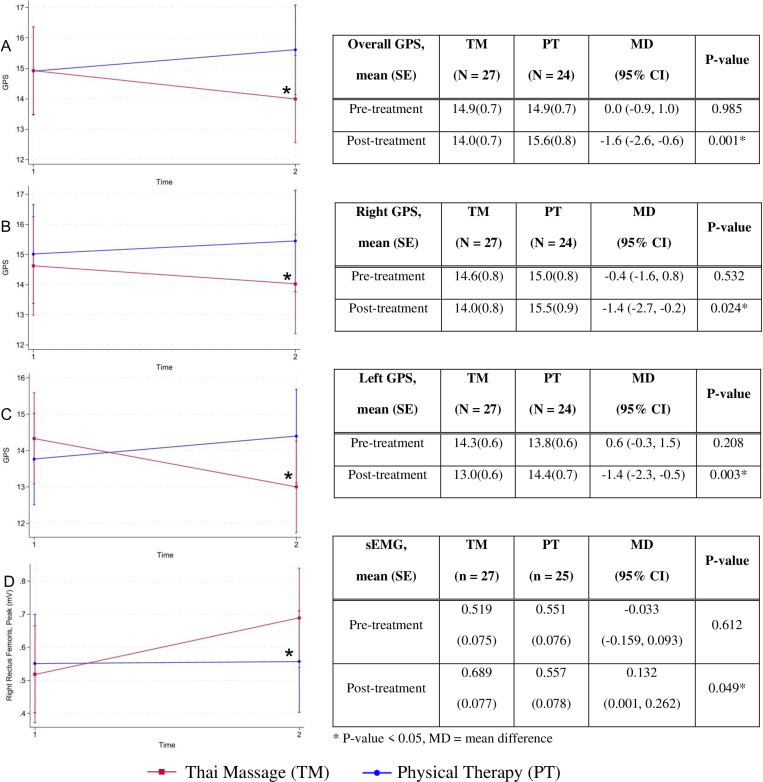
The outcomes categorized into Thai Massage (TM) and Physical Therapy (PT). **(A)** Overall Gait Profile Score (GPS), **(B)** Right GPS, **(C)** Left GPS, **(D)** Peak activity of right rectus femoris muscle from surface electromyography (sEMG).

The treatment effects were estimated using mixed-effects model in consideration of randomization groups and visits. TM significantly decreased overall GPS (MD -1.6 (95%CI -2.6, -0.6), *P*-value = 0.001), right GPS (MD -1.4 (95%CI -2.7, -0.2), *P*-value = 0.024), and left GPS (MD -1.4 (95%CI -2.3, -0.5), *P*-value = 0.003) when compared to PT, Fig 3. Additionally, TM significantly increased the right rectus femoris peak activity when compared to PT (MD 0.132 mV (95%CI 0.001, 0.262), *P*-value = 0.049). No significant differences of ROM, muscle tone, other sEMG parameters, foot pressure, and VO_2_max were detected between group A and group B, and between TM and PT (S6-10 Tables in S1 File).

After adjusted for possible confounding factors (hospital physical therapy/home video program, ankle dorsiflexion at knee extension and at knee flexion), TM was superior to PT in right, left, and overall GPS, and right rectus femoris peak muscle activity, Table 4. At the end of the study, patient compliance exceeded 80% without any complication.

**Table 4:** The adjusted outcomes comparing Thai massage to physical therapy by using multilevel mixed-effects linear regression models.

Adjusted outcomes, mean (SE)	Thai Massage (N = 27)	Physical Therapy (N = 24)	Mean Difference (95% confidence interval)	*P*-value
**Overall GPS**				
Pre-treatment	14.8 (0.7)	14.8 (0.7)	0.0 (-1.0, 1.0)	0.991
Post-treatment	13.9 (0.7)	15.5 (0.7)	-1.6 (-2.6, -0.6)	0.001*
**Right GPS**				
Pre-treatment	14.5 (0.8)	14.9 (0.8)	-0.4 (-1.6, 0.8)	0.507
Post-treatment	14.0 (0.8)	15.3 (0.9)	-1.4 (-2.7, -0.1)	0.029*
**Left GPS**				
Pre-treatment	14.3 (0.6)	13.7 (0.6)	0.6 (-0.3, 1.4)	0.219
Post-treatment	12.9 (0.6)	14.4 (0.6)	-1.5 (-2.4, -0.6)	0.001*
**sEMG of right rectus femoris muscle, peak (mV)**	N = 27	N = 25		
Pre-treatment	0.521 (0.072)	0.550 (0.073)	-0.028 (-0.154, 0.097)	0.656
Post-treatment	0.686 (0.074)	0.540 (0.076)	0.146 (0.014, 0.278)	0.030*

Adjusted by hospital physical therapy/home video program, left ankle dorsiflexion at knee extension and at knee flexion, SE = standard error, sEMG = surface electromyography, * significant *P*-value < 0.05.

## Discussion

Massage enables blood flow, muscle stretching, and eliminate lactic acidosis from muscles [16]. These mechanisms may prevent contracture, facilitate muscle function and walking abilities among CP patients. Our study stands out as the pioneer to incorporate TM into CP treatment compared to standard PT. This RCT explored gait performance between 6-week TM and standard PT among ambulatory individuals with spastic CP. In comparison to PT, TM revealed significant lower GPS (MD -1.4 for the right, -1.5 for the left, and -1.6 for the overall) and higher peak activity of the right rectus femoris (0.146). These findings suggested that TM gait patterns were more likely resembled those of the normal reference population than the PT group [26].

Over the years, numerous studies have assessed the efficacy of different massage modalities in CP [20]. Combining passive muscle stretching and whole body vibration helped to improve spasticity, muscle strength, proprioception, and balance [7]. From an RCT involving 20 CP children, Swedish massage could diminish spasticity measured by the MAS, as well as enhance ROM and gross motor function [18]. Additionally, the massage group improved scores in cognition, social skills, and dressing according to the developmental profile. During face-to-face play interaction, the Swedish massage group displayed more positive facial expressions and reduced limb activity. Mahmood et al. [19] conducted an RCT comparing traditional Pakistani massage combining with PT to conventional PT in 75 spastic CP patients. After 6 and 12 weeks of intervention, muscle tone (MAS) significantly reduced without significantly ameliorated GMFM [19]. In the realm of TM, the case series (17 spastic CP patients) and the RCT (13 spastic diplegic CP patients) from Malila et al. [5,23] reported significantly diminished MAS, especially quadriceps muscles after a 30-minute session.

Currently, the TM encompasses various classifications. The CTTM seamlessly integrates ancient Thai wisdom with scientific substantiation, and set it apart from other massage techniques. Distinctive features have a potential to enhance walking ability and overall quality of life in individuals with CP. Using only fingers and palms to apply pressure and knead along basic massage lines (BMLs) and specific major signal points (MaSPs) could restore energy flow and stimulate blood circulation [31]. Furthermore, Plakornkul et al. [32] and Viravud et al. [33] empirical supported CTTM effects on nerves, blood and heat regulation. The anatomical of open the wind gait via MaSPs correlated with blood flow and skin temperature in the upper [32] and especially lower extremities [33]. This principle is believed to ease the pain, muscle stiffness, and improve psychological well-being [31].

Spasticity reduction seemed to be benefited from TM [5,23] The percentage of normal MAS in this study increased after treatments (S1 and S7 Tables in S1 File) but the effects of TM on MAS, foot pressure, and VO_2_max did not overcome those of PT. Aside from the MAS advantage, specific CTTM promoted rectus muscle activity and walking ability among CP patients. The significant level of TM effects on the right rectus femoris peak activity when compared to PT may occur by chance (MD 0.132 mV, (95%CI 0.001, 0.262), *P*-value = 0.049) since its P-value was nearly 0.05, and the lower border of 95%CI was also closed to zero. It was worth noting that baseline left ankle dorsiflexion with knee extension and flexion was higher in the randomization group B (PT-TM) than group A (TM-PT), Table 3. After adjusting these factors, the peak activity of the right rectus femoris muscle and the means of GPS (overall, right, and left) among TM were significantly superior to PT (Table 4). The higher peak activity observed in the right rectus femoris of the TM group may attribute to increased muscle engagement [34]. This 30-mimute TM twice a week for a 6-week duration adequately lowering overall GPS, MD -1.6 (95%CI -2.6, -0.6) when compared to PT. Differences of GPS between both interventions (MD 1.4–1.6, Table 4) nearly reach a clinically significant of MCID 1.7 [26]. While the previous trial demonstrated insignificant one-minute walking test between single TM session and passive static stretching [23] with estimated MD -8.81 m (95%CI -28.8, 11.2).

The GPS of both treatments slightly improved in Phase I. At Phase II, it was obviously enhanced in TM but deteriorated in PT. The plausible explanations are inadequate PT duration, incomplete follow-up, and measurement artefacts. From the literature review, PT duration for CP was performed 30–60 minutes, 2–7 times per week for 6–32 weeks [8,9,19,35–37]. A meta-analysis found daily hours and duration of training program for CP positively correlated with GMFM [38]. Our study applied the 30-minute session, 2 times per week for 6 weeks. With this low intensity and short duration of PT, its actual effects may insufficiently appear. Moreover, only 11 (68.75%) out of initially assigned 16 patients in group A (TM-PT) completed PT at follow-up in Phase II, and might not represent the PT outcome (attrition bias). Small sample size and kinematic assessment methods for GPS (electrode interface/cable irregularity) [34] inevitably led to random and measurement errors in both intervention groups.

Our research design specified a washout period of 6 weeks, referencing the study conducted by Cheng et al. [39], and the duration of muscle stiffness development after cerebral ischemia [40]. Also, the multilevel mixed-effects linear regression model indicated the absence of any carry-over effect between 6 and 12 weeks. Both interventions in this study can be administered either at the hospital by our proficient traditional Thai medicine practitioners and physiotherapists, or at home by caregivers through our instructional video. After completing intervention, the compliances were more than 80%. This method is comparable to the other caregivers home-based program for CP [37,41]. In Pakistan, combined training (exercise and pediatric massage) under supervision with video recording practically decreased MAS after 6 weeks and GMFCS after 12 weeks [37]. The intensive infant massage program for high-risk CP (duration 20 minutes, 5 times/week for 8 weeks) in Tuscany, Italy achieved mothers’ compliance up to 89.47% [41]. For clinical application, both TM and PT effectively improves muscle and walking ability and could be applied in CP patients. In regards with resource scarcity [24,37], delivery methods might be shifted from the hospital to home-based program where appropriate.

This study has several strengths. Notably, the cross-over single-blinded randomized controlled trial with concealment, good compliance, no carry-over effects, and intention-to-treat analysis conveys research validity. For precise and unbiased results, an innovative approach by utilizing 3D gait analysis, and the objective measurements of sEMG, foot pressure, and VO_2_max were applied. On the other hand, certain limitations should be acknowledged. Firstly, the relatively small sample size may impact a clinically significant detection between treatments. Secondly, incomplete follow-up more than 20% affected outcome attrition. Thirdly, low intensity and short duration of PT insufficiently revealed its actual effects. Finally, adherence of patients’ guardians, who opted for both interventions based on our video demonstration, posed a challenge. However, neither complication, nor cointervention was detected and the patient’s compliance were more than 80%.

## Conclusion

According to ambulatory CP patients, TM significantly improved gait performance (GPS) and rectus femoris peak activity than PT without complications. In contrast, its effects on MAS, foot pressure, and VO_2_max could not overcome those of PT. This implies that TM could be considered as an alternative option for treating spastic CP in children, particularly in PT scarcity areas. With 30-minute session, 2 times per week for 6 weeks, TM may be practically applied for healthcare sectors and family to enhance patients’ walking ability. Nevertheless, the results from this study had some limitations from small samples, measurement artefacts, and short duration of PT intervention. Also, both treatments showed insignificantly different outcomes in each solitary phase. Conducting a large-scale non-inferiority trial is advised to obtain more comprehensive information about its effectiveness.

## Supporting information

S1 File **S1 Table. Baseline muscle tone by modified Ashworth scale (MAS).** Other baseline characteristics, clinical examination, motions, gait parameters, muscle tone, sEMG, GPS, foot pressure, and VO_2_max of both groups demonstrated similar patterns. **S2 Table. Baseline temporal spatial parameters.** Other baseline characteristics, clinical examination, motions, gait parameters, muscle tone, sEMG, GPS, foot pressure, and VO_2_max of both groups demonstrated similar patterns. **S3 Table. Baseline surface electromyography.** Other baseline characteristics, clinical examination, motions, gait parameters, muscle tone, sEMG, GPS, foot pressure, and VO_2_max of both groups demonstrated similar patterns. **S4 Table. Baseline Gait Profile Score, foot pressure, and maximum rate of oxygen consumption (VO**_**2**_**max).** Other baseline characteristics, clinical examination, motions, gait parameters, muscle tone, sEMG, GPS, foot pressure, and VO_2_max of both groups demonstrated similar patterns. **S5 Table. Mixed-effects regression with post-hoc Bonferroni test comparing between randomization groups** Overall GPS, right and left GPS, and right rectus femoral peak activity showed insignificant differences between randomization groups. **S6 Table. Post-treatment range of motion (ROM) for Thai massage and physical therapy.** No significant differences of ROM, muscle tone, other sEMG parameters, foot pressure, and VO_2_max were detected between group A and group B, and between TM and PT. **S7 Table. Post-treatment muscle tone for Thai massage and physical therapy** No significant differences of ROM, muscle tone, other sEMG parameters, foot pressure, and VO_2_max were detected between group A and group B, and between TM and PT. **S8 Table. Post-treatment temporal spatial parameters for Thai massage and physical therapy.** No significant differences of ROM, muscle tone, other sEMG parameters, foot pressure, and VO_2_max were detected between group A and group B, and between TM and PT. **S9 Table. Post-treatment surface electromyography for Thai massage and physical therapy.** No significant differences of ROM, muscle tone, other sEMG parameters, foot pressure, and VO_2_max were detected between group A and group B, and between TM and PT**. S10 Table. Post-treatment foot pressure and oxygen consumption for Thai massage and physical therapy.** No significant differences of ROM, muscle tone, other sEMG parameters, foot pressure, and VO_2_max were detected between group A and group B, and between TM and PT.
(DOCX)

S1 Protocol Trial study protocol (English translation). (DOCX)

S2 Protocol Trial study protocol (Thai original). (DOCX)

S1 CONSORT checklist CONSORT checklist of information to include when reporting a randomised trial. (DOC)

S1 Full data set Full data set. (XLSX)
