# Supplementary material for: The effect of traditional Thai massage vs routine physical therapy on gait pattern in spastic cerebral palsy: A cross-over randomized controlled trial
Source: PLoS One. 2025 May 29;20(5):e0325169. doi: 10.1371/journal.pone.0325169 (PMC12122028; doi:10.1371/journal.pone.0325169)
Supplement: S1 File — S1 Table. Baseline muscle tone by modified Ashworth scale (MAS). Other baseline characteristics, clinical examination, motions, gait parameters, muscle tone, sEMG, GPS, foot pressure, and VO2max of both groups demonstrated similar patterns. S2 Table. Baseline temporal spatial parameters. Other baseline characteristics, clinical examination, motions, gait parameters, muscle tone, sEMG, GPS, foot pressure, and VO2max of both groups demonstrated similar patterns. S3 Table. Baseline surface electromyography. Other baseline characteristics, clinical examination, motions, gait parameters, muscle tone, sEMG, GPS, foot pressure, and VO2max of both groups demonstrated similar patterns. S4 Table. Baseline Gait Profile Score, foot pressure, and maximum rate of oxygen consumption (VO2max). Other baseline characteristics, clinical examination, motions, gait parameters, muscle tone, sEMG, GPS, foot pressure, and VO2max of both groups demonstrated similar patterns. S5 Table. Mixed-effects regression with post-hoc Bonferroni test comparing between randomization groups Overall GPS, right and left GPS, and right rectus femoral peak activity showed insignificant differences between randomization groups. S6 Table. Post-treatment range of motion (ROM) for Thai massage and physical therapy. No significant differences of ROM, muscle tone, other sEMG parameters, foot pressure, and VO2max were detected between group A and group B, and between TM and PT. S7 Table. Post-treatment muscle tone for Thai massage and physical therapy No significant differences of ROM, muscle tone, other sEMG parameters, foot pressure, and VO2max were detected between group A and group B, and between TM and PT. S8 Table. Post-treatment temporal spatial parameters for Thai massage and physical therapy. No significant differences of ROM, muscle tone, other sEMG parameters, foot pressure, and VO2max were detected between group A and group B, and between TM and PT. S9 Table. Post-treatment surface electromyography for [file pone.0325169.s001.docx]

**Supplementary Tables**

**S1 Table.** Baseline muscle tone by modified Ashworth scale (MAS)

| **Normal muscle tone (MAS = 0), %** | **Group A (TM-PT)**  **N = 16** | **Group B (PT-TM)**  **N = 16** | **P-value** |
| --- | --- | --- | --- |
| **Right** | | | |
| Iliopsoas | 12 (75.0) | 8 (50.0) | 0.213 |
| Gluteus maximus | 6 (37.5) | 9 (56.3) | 0.881 |
| Hip adductor | 4 (25.0) | 7 (43.8) | 0.237 |
| Rectus femoris | 6 (37.5) | 5 (31.3) | 0.923 |
| Hamstring | 5 (31.3) | 6 (37.5) | 1.000 |
| Tibialis anterior | 15 (93.8) | 15 (93.8) | 1.000 |
| Gastrocnemius | 2 (12.5) | 1 (6.3) | 0.776 |
| Tibialis posterior | 6 (37.5) | 4 (25.0) | 0.313 |
| Soleus | 2 (12.5) | 1 (6.3) | 0.286 |
| Peroneus | 13 (81.3) | 14 (87.5) | 1.000 |
| **Left** | | | |
| Iliopsoas | 12 (75.0) | 9 (56.3) | 0.171 |
| Gluteus maximus | 6 (37.5) | 10 (62.5) | 0.659 |
| Hip adductor | 4 (25.0) | 8 (50) | 0.335 |
| Rectus femoris | 4 (25.0) | 6 (37.5) | 0.895 |
| Hamstring | 4 (25.0) | 8 (50) | 0.612 |
| Tibialis anterior | 15 (93.8) | 15 (93.8) | 1.000 |
| Gastrocnemius | 2 (12.5) | 3 (18.8) | 0.346 |
| Tibialis posterior | 7 (43.8) | 7 (43.8) | 0.881 |
| Soleus | 2 (12.5) | 3 (18.8) | 0.912 |
| Peroneus | 12 (75.0) | 14 (87.5) | 0.496 |

TM = Thai massage, PT = physiotherapy

**S2 Table.** Baseline temporal spatial parameters

| **Temporal spatial parameters, mean (SD)** | **Group A (TM-PT)**  **N = 15)** | **Group B (PT-TM)**  **N = 15** | **P-value** |
| --- | --- | --- | --- |
| Velocity (cm/s) | 64.3 (25.8) | 74.8 (30.0) | 0.302 |
| Cadence (step/min) | 96.2 (24.5) | 104.2 (24.8) | 0.385 |
| Stride length (cm) | 76.9 (24.3) | 82.9 (20.7) | 0.471 |
| Step width (cm) | 12.4 (5.4) | 12.5 (4.5) | 0.939 |

TM = Thai massage, PT = physiotherapy

**S3 Table.** Baseline surface electromyography

| **Electromyography (mV),**  **mean (SD)** | **Group A (TM-PT)**  **N = 16** | **Group B (PT-TM)**  **N = 16)** | **P-value** |
| --- | --- | --- | --- |
| **Right** | | | |
| Gluteus medius, root mean square | 0.154 (0.137) | 0.192 (0.124) | 0.152 |
| Gluteus medius, peak | 0.592 (0.459) | 0.798 (0.418) | 0.083 |
| Rectus femoris, root mean square | 0.139 (0.095) | 0.136 (0.100) | 0.970 |
| Rectus femoris, peak | 0.571 (0.425) | 0.582 (0.433) | 0.498 |
| Biceps, root mean square | 0.105 (0.060) | 0.103 (0.064) | 0.940 |
| Biceps, peak | 0.403 (0.273) | 0.387 (0.278) | 0.880 |
| Gastrocnemius, root mean square | 0.128 (0.072) | 0.189 (0.266) | 0.163 |
| Gastrocnemius, peak | 0.491 (0.315) | 0.579 (0.304) | 0.163 |
| Tibialis anterior, root mean square | 0.147 (0.106) | 0.124 (0.044) | 0.763 |
| Tibialis anterior, peak | 0.520 (0.389) | 0.434 (0.221) | 0.474 |
| **Left** | | | |
| Gluteus medius, root mean square | 0.137 (0.084) | 0.186 (0.138) | 0.346 |
| Gluteus medius, peak | 0.526 (0.329) | 0.788 (0.523) | 0.113 |
| Rectus femoris, root mean square | 0.117 (0.073) | 0.149 (0.098) | 0.598 |
| Rectus femoris, peak | 0.448 (0.316) | 0.635 (0.431) | 0.327 |
| Biceps, root mean square | 0.140 (0.142) | 0.118 (0.064) | 0.735 |
| Biceps, peak | 0.455 (0.362) | 0.443 (0.250) | 0.274 |
| Gastrocnemius, root mean square | 0.141 (0.081) | 0.146 (0.077) | 0.598 |
| Gastrocnemius, peak | 0.560 (0.334) | 0.599 (0.357) | 0.429 |
| Tibialis anterior, root mean square | 0.129 (0.070) | 0.129 (0.066) | 0.679 |
| Tibialis anterior, peak | 0.466 (0.323) | 0.474 (0.285) | 0.327 |

TM = Thai massage, PT = physiotherapy

**S4 Table.** Baseline Gait Profile Score, foot pressure, and maximum rate of oxygen consumption (VO_2_max)

| **Variables, mean (SD)** | **Group A (TM-PT)**  **N = 15** | **Group B (PT-TM)**  **N = 15** | **P-value** |
| --- | --- | --- | --- |
| **Gait Profile Score** |  |  |  |
| Left | 14.0 (4.2) | 14.0 (3.1) | 0.985 |
| Right | 15.0 (4.7) | 15.0 (4.5) | 0.994 |
| Overall | 15.0 (4.4) | 14.8 (3.8) | 0.929 |
| **Foot pressure (gm/cm^2^)** |  |  |  |
| **Right** |  |  |  |
| Static | 292.2 (217.6) | 221.1 (75.0) | 0.320 |
| Dynamic | 1488.8 (763.1) | 1438.2 (648.3) | 0.468 |
| **Left** |  |  |  |
| Static | 297.1 (161.0) | 262.3 (85.9) | 0.852 |
| Dynamic | 1279.3 (320.6) | 1419.1 (579.8) | 0.423 |
| **Oxygen consumption** | **N = 14** | **N = 14** |  |
| VO_2_ max (ml/min/kg) | 23.3 (5.2) | 22.8 (8.3) | 0.847 |

TM = Thai massage, PT = physiotherapy

**S5 Table.** Mixed-effects regression with post-hoc Bonferroni test comparing between randomization groups

| **Variables** | **Mean (95% confidence interval)** | | |
| --- | --- | --- | --- |
|  | **Group A (TM-PT)** | **Group B (PT-TM)** | **Group B – Group A** |
| **Left Gait Profile Score** |  |  |  |
| - Visit 1 (baseline) | 14.17 (12.44, 15.91) | 14.05 (12.28, 15.82) | -0.13 (-4.08, 3.82) |
| - Visit 2 (1^st^ intervention) | 13.19 (11.43, 14.95) | 14.33 (12.51, 16.15) | 1.14 (-2.90, 5.17) |
| - Visit 3 (baseline) | 13.42 (11.61, 15.23) | 14.47 (12.65, 16.29) | 1.05 (-3.04, 5.14) |
| - Visit 4 (2^nd^ intervention) | 14.66 (12.80, 16.53) | 12.78 (10.96, 14.60) | -1.89 (-6.04, 2.27) |
| - Visit 2 – Visit 1 | -0.98 (-2.91, 0.95) | -0.28 (-2.66, 2.23) |  |
| - Visit 3 – Visit 1 | -0.76 (-2.76, 1.24) | 0.42 (-1.52, 2.37) |  |
| - Visit 4 – Visit 1 | 0.49 (-1.64, 2.62) | -1.27 (-3.21, 0.67) |  |
| - Visit 3 – Visit 2 | 0.22 (-1.78, 2.23) | 0.14 (-1.82, 2.10) |  |
| - Visit 4 – Visit 2 | 1.47 (-0.67, 3.61) | -1.55 (-3.51, 0.40) |  |
| - Visit 4 – Visit 3 | 1.25 (-0.92, 3.41) | -1.69 (-3.65, -0.26) |  |
| **Right Gait Profile Score** |  |  |  |
| - Visit 1 (baseline) | 15.34 (13.11, 17.56) | 15.03 (12.77, 17.30) | -0.31 (-5.36, 4.75) |
| - Visit 2 (1^st^ intervention) | 15.05 (12.80, 17.31) | 14.23 (11.90, 16.57) | -0.82 (-5.99, 4.35) |
| - Visit 3 (baseline) | 14.79 (12.46, 17.11) | 13.74 (11.41, 16.07) | -1.05 (-6.29, 4.20) |
| - Visit 4 (2^nd^ intervention) | 16.62 (14.22, 19.03) | 12.83 (10.49, 15.16) | -3.80 (-9.14, 1.54) |
| - Visit 2 – Visit 1 | -0.29 (-2.89, 2.32) | -0.80 (-3.42, 1.82) |  |
| - Visit 3 – Visit 1 | -0.55 (-3.25, 2.14) | -1.29 (-3.91, 1.33) |  |
| - Visit 4 – Visit 1 | 1.29 (-1.59, 4.16) | -2.21 (-4.83, 0.41) |  |
| - Visit 3 – Visit 2 | -0.27 (-2.97, 2.44) | -0.49 (-3.13, 2.15) |  |
| - Visit 4 – Visit 2 | 1.57 (-1.31, 4.46) | -1.41 (-4.05, 1.23) |  |
| - Visit 4 – Visit 3 | 1.84 (-1.08, 4.76) | -0.92 (-3.56, 1.73) |  |

**S5 Table.** Mixed-effects regression with post-hoc Bonferroni test comparing between randomization groups (Continued)

| **Variables** | **Mean (95% confidence interval)** | | |
| --- | --- | --- | --- |
|  | **Group A (TM-PT)** | **Group B (PT-TM)** | **Group B – Group A** |
| **All Gait Profile Score** |  |  |  |
| - Visit 1 (baseline) | 15.27 (13.30, 17.24) | 14.84 (12.83, 16.85) | -0.43 (-4.92, 4.05) |
| - Visit 2 (1^st^ intervention) | 14.91 (12.92, 16.91) | 14.82 (12.75, 16.87) | -0.10 (-4.67, 4.47) |
| - Visit 3 (baseline) | 14.86 (12.81, 16.91) | 14.48 (12.42, 16.54) | -0.39 (-5.01, 4.24) |
| - Visit 4 (2^nd^ intervention) | 16.46 (14.36, 18.56) | 12.97 (10.91, 15.03) | -3.49 (-8.18, 1.21) |
| - Visit 2 – Visit 1 | -0.36 (-2.42, 1.71) | -0.02 (-2.10, 2.05) |  |
| - Visit 3 – Visit 1 | -0.41 (-2.54. 1.72) | -0.36 (-2.43, 1.71) |  |
| - Visit 4 – Visit 1 | 1.18 (-1.09, 3.46) | -1.87 (-3.94, 0.20) |  |
| - Visit 3 – Visit 2 | -0.05 (-2.19, 2.09) | -0.34 (-2.42, 1.75) |  |
| - Visit 4 – Visit 2 | 1.54 (-0.74, 3.82) | -1.85 (-3.93, 0.24) |  |
| - Visit 4 – Visit 3 | 1.59 (-0.72, 3.90) | -1.51 (-3.59, 0.58) |  |
| **Right rectus femoris, peak** |  |  |  |
| - Visit 1 (baseline) | 0.50 (0.30, 0.70) | 0.61 (0.41, 0.81) | 0.11 (-0.34, 0.56) |
| - Visit 2 (1^st^ intervention) | 0.79 (0.58, 1.00) | 0.51 (0.31, 0.72) | -0.28 (-0.74, 0.19) |
| - Visit 3 (baseline) | 0.47 (0.26, 0.69) | 0.54 (0.33, 0.74) | 0.07 (-0.41, 0.54) |
| - Visit 4 (2^nd^ intervention) | 0.58 (0.35, 0.80) | 0.57 (0.36, 0.79) | -0.00 (-0.49. 0.49) |
| - Visit 2 – Visit 1 | 0.29 (0.03, 0.55) | -0.10 (-0.36, 0.16) |  |
| - Visit 3 – Visit 1 | -0.03 (-0.30, 0.25) | -0.07 (-0.33, 0.19) |  |
| - Visit 4 – Visit 1 | 0.08 (-0.22, 0.37) | -0.03 (-0.30, 0.23) |  |
| - Visit 3 – Visit 2 | -0.32 (-0.60, -0.04) | 0.02 (-0.24, 0.29) |  |
| - Visit 4 – Visit 2 | -0.21 (-0.51, 0.08) | 0.06 (-0.21, 0.33) |  |
| - Visit 4 – Visit 3 | 0.10 (-0.20, 0.41) | 0.04 (-0.23, 0.31) |  |

**S6 Table.** Post-treatment range of motion (ROM) for Thai massage and physical therapy

| **ROM (degrees),**  **mean (SE)** | **Right side** | | | **Left side** | | |
| --- | --- | --- | --- | --- | --- | --- |
|  | **Thai Massage**  **(N = 27)** | **Physical Therapy**  **(N = 25)** | **P-value** | **Thai Massage**  **(N = 27)** | **Physical Therapy**  **(N = 25)** | **P-value** |
| Hip flexion | 125.1 (2.2) | 125.8 (2.2) | 0.725 | 126 (2.6) | 122.1 (2.6) | 0.195 |
| Hip extension | 21.5 (1.0) | 22.4 (1.1) | 0.457 | 21.0 (2.3) | 22.3 (2.4) | 0.676 |
| Hip abduction | 38.8 (1.3) | 40.7 (1.4) | 0.168 | 39.1 (1.4) | 40.7 (1.5) | 0.286 |
| Hip adduction | 24.6 (1.1) | 26.3 (1.2) | 0.238 | 24.1 (1.3) | 24.4 (1.4) | 0.874 |
| Hip internal rotation | 66.4 (2.2) | 66.2 (2.3) | 0.891 | 65.3 (2.2) | 66.2 (2.2) | 0.694 |
| Hip external rotation | 56.7 (3.0) | 58.9 (3.0) | 0.367 | 57.9 (3.4) | 59.2 (3.5) | 0.726 |
| Knee flexion | 140.4 (2.0) | 141.9 (2.1) | 0.594 | 140.6 (1.3) | 142.3 (1.3) | 0.218 |
| Knee extension | -1.8 (1.3) | -1.5 (1.3) | 0.675 | -0.6 (1.1) | -0.5 (1.1) | 0.858 |
| Ankle dorsiflexion at KE | 8.9 (2.3) | 9.2 (2.3) | 0.854 | 7.1 (2.0) | 7.4 (2.0) | 0.794 |
| Ankle dorsiflexion at KF | 15.3 (1.9) | 14.9 (1.9) | 0.763 | 14.3 (1.9) | 15.0 (2.0) | 0.657 |
| Ankle plantar flexion | 71.3 (1.9) | 70.2 (1.9) | 0.574 | 71.6 (2.0) | 71.4 (2.0) | 0.920 |
| Ankle inversion | 50.4 (2.3) | 53.3 (2.4) | 0.168 | 51.5 (2.2) | 52.4 (2.3) | 0.667 |
| Ankle eversion | 37.2 (2.3) | 36.9 (2.4) | 0.924 | 38.2 (2.0) | 38.0 (2.1) | 0.935 |
| Popliteal angle | 42.0 (2.3) | 41.3 (2.3) | 0.704 | 39.3 (2.1) | 41.7 (2.1) | 0.184 |

SE = standard error

**S7 Table.** Post-treatment muscle tone for Thai massage and physical therapy

| **Normal muscle tone, n (%)** | **Thai Massage**  **(N = 27)** | **Physical Therapy**  **(N = 25)** | **RR  (95% CI)** | **P-value** |
| --- | --- | --- | --- | --- |
| **Right** | | | | |
| Iliopsoas | 17 (63.0) | 20 (80.0) | 0.80 (0.41, 1.53) | 0.493 |
| Gluteus maximus | 11 (40.7) | 15 (60.0) | 0.70 (0.32, 1.54) | 0.373 |
| Hip adductor | 11 (40.7) | 12 (48.0) | 0.89 (0.39, 2.05) | 0.782 |
| Rectus femoris | 7 (25.9) | 11 (44.0) | 0.57 (0.22, 1.46) | 0.239 |
| Hamstring | 7 (25.9) | 11 (44.0) | 0.57 (0.22, 1.46) | 0.238 |
| Tibialis anterior | 27 (100.0) | 25 (100.0) | 1.00 (0.58, 1.73) | 1.000 |
| Gastrocnemius | 3 (11.1) | 2 (8.0) | 1.37 (0.22, 8.57) | 0.734 |
| Tibialis posterior | 8 (29.6) | 4 (16.0) | 1.78 (0.54, 5.90) | 0.347 |
| Soleus | 3 (11.1) | 4 (16.0) | 0.68 (0.15, 3.07) | 0.615 |
| Peroneus | 24 (88.9) | 24 (96.0) | 0.89 (0.50, 1.57) | 0.683 |
| **Left** | | | | |
| Iliopsoas | 16 (59.3) | 21 (84.0) | 0.71 (0.37, 1.37) | 0.309 |
| Gluteus maximus | 10 (37.0) | 15 (60.0) | 0.63 (0.28, 1.43) | 0.273 |
| Hip adductor | 12 (44.4) | 14 (56.0) | 0.82 (0.37, 1.80) | 0.621 |
| Rectus femoris | 10 (37.0) | 11 (44.0) | 0.81 (0.34, 1.90) | 0.625 |
| Hamstring | 8 (29.6) | 9 (36.0) | 0.79 (0.30, 2.05) | 0.628 |
| Tibialis anterior | 27 (100.0) | 25 (100.0) | 1.00 (0.58, 1.73) | 1.000 |
| Gastrocnemius | 3 (11.1) | 5 (20.0) | 0.54 (0.13, 2.30) | 0.405 |
| Tibialis posterior | 9 (33.3) | 11 (44.0) | 0.80 (0.33, 1.97) | 0.627 |
| Soleus | 5 (18.5) | 5 (20.0) | 0.93 (0.26, 3.26) | 0.906 |
| Peroneus | 24 (88.9) | 24 (96.0) | 0.93 (0.52, 1.64) | 0.797 |

SE = standard error, RR = Relative Risk

**S8 Table.** Post-treatment temporal spatial parameters for Thai massage and physical therapy

| **Temporal spatial parameters, mean (SE)** | **Thai Massage**  **(N = 27)** | **Physical Therapy**  **(N = 24)** | **P-value** |
| --- | --- | --- | --- |
| Velocity (cm/s) | 66.3 (4.9) | 71.6 (5.0) | 0.107 |
| Cadence (step/min) | 107.0 (5.0) | 106.5 (5.2) | 0.892 |
| Stride length (cm) | 78.0 (4.0) | 81.5 (4.1) | 0.065 |
| Step width (cm) | 11.6 (0.8) | 12.4 (0.8) | 0.090 |

SE = standard error

**S9 Table.** Post-treatment surface electromyography for Thai massage and physical therapy

| **Electromyography (mV), mean (SE)** | **Thai Massage**  **(N = 27)** | **Physical Therapy**  **(N = 25)** | **P-value** |
| --- | --- | --- | --- |
| **Right** | | | |
| Gluteus medius, root mean square | 0.185 (0.024) | 0.160 (0.025) | 0.270 |
| Gluteus medius, peak | 0.691 (0.082) | 0.650 (0.085) | 0.604 |
| Rectus femoris, root mean square | 0.159 (0.017) | 0.142 (0.018) | 0.249 |
| Rectus femoris, peak | 0.689 (0.077) | 0.557 (0.078) | 0.049* |
| Biceps, root mean square | 0.101 (0.011) | 0.100 (0.012) | 0.962 |
| Biceps, peak | 0.369 (0.050) | 0.388 (0.051) | 0.054 |
| Gastrocnemius, root mean square | 0.142 (0.037) | 0.136 (0.038) | 0.904 |
| Gastrocnemius, peak | 0.569 (0.057) | 0.508 (0.059) | 0.318 |
| Tibialis anterior, root mean square | 0.151 (0.015) | 0.128 (0.015) | 0.212 |
| Tibialis anterior, peak | 0.548 (0.058) | 0.467 (0.060) | 0.269 |
| **Left** | | | |
| Gluteus medius, root mean square | 0.143 (0.021) | 0.170 (0.022) | 0.194 |
| Gluteus medius, peak | 0.605 (0.082) | 0.659 (0.084) | 0.467 |
| Rectus femoris, root mean square | 0.131 (0.016) | 0.136 (0.016) | 0.686 |
| Rectus femoris, peak | 0.558 (0.070) | 0.500 (0.071) | 0.292 |
| Biceps, root mean square | 0.116 (0.020) | 0.132 (0.021) | 0.499 |
| Biceps, peak | 0.427 (0.056) | 0.461 (0.057) | 0.580 |
| Gastrocnemius, root mean square | 0.135 (0.014) | 0.150 (0.014) | 0.233 |
| Gastrocnemius, peak | 0.557 (0.062) | 0.628 (0.064) | 0.255 |
| Tibialis anterior, root mean square | 0.141 (0.017) | 0.160 (0.018) | 0.418 |
| Tibialis anterior, peak | 0.570 (0.070) | 0.557 (0.073) | 0.891 |

SE = standard error * Statistically significant difference (P-value < 0.05)

**S10 Table.** Post-treatment foot pressure and oxygen consumption for Thai massage and physical therapy

| **Foot pressure, mean (SE)** | **Thai Massage**  **(N = 26)** | **Physical Therapy**  **(N = 24)** | **P-value** |
| --- | --- | --- | --- |
| **Right** | | | |
| Static (gm/cm^2^) | 245.4 (27.5) | 229.5 (28.4) | 0.347 |
| Dynamic (gm/cm^2^) | 1365.0 (121.9) | 1314.8 (125.8) | 0.786 |
| **Left** | | | |
| Static (gm/cm^2^) | 268.0 (21.9) | 291.0 (22.6) | 0.347 |
| Dynamic (gm/cm^2^) | 1301.9 (99.3) | 1272.9 (102.1) | 0.786 |
| **Oxygen consumption,**  **mean (SE)** |  |  |  |
| VO_2_ max (ml/min/kg) | 21.5 (1.4) | 20.3 (1.5) | 0.386 |

SE = standard error
