# Supplementary material for: The effect of traditional Thai massage vs routine physical therapy on gait pattern in spastic cerebral palsy: A cross-over randomized controlled trial
Source: PLoS One. 2025 May 29;20(5):e0325169. doi: 10.1371/journal.pone.0325169 (PMC12122028; doi:10.1371/journal.pone.0325169)
Supplement: S1 Protocol — (DOCX) [file pone.0325169.s002.docx]

# Research Project Proposal Form for Consideration by the Human Research Ethics Committee, Faculty of Medicine, Ramathibodi Hospital, Mahidol University (Full Version)

1. **Project Name**

The effect of traditional Thai massage vs. routine physical therapy on gait pattern in spastic cerebral palsy

# Name of the Head of the Research Project

Peerapat Lertwiram

# Collaborators

Patarawan Woratanarat

Chanika Angsanuntsukh

Apiphan Iamchaimongkol

Suchanont Baosuwan

Ponsaphat Ongtanasin

Phimpisut Srinorasit

Krongkaew Klaewkasikum

Tanyaporn Patathong

# Introduction, Principles and Rationale

Cerebral palsy is a syndrome in which the body is abnormal in posture and movement, which is caused by a static brain pathology during the growth and development of the brain. Cerebral palsy is commonly found in developed countries with the incidence of 7 per 1000 livebirths.^1^

The causes of cerebral palsy can occur during pregnancy, such as high blood pressure, infection, exposure to toxic substances, heart and respiratory diseases, as well as metabolic diseases. Perinatal causes are placenta previa, prolonged labor, umbilical cord prolapse, abnormal fetal presentation, cephalopelvic disproportion, and preterm delivery. And postpartum period cases are such as head injury, brain vessel disorders, illnesses that cause cerebral ischemia or oxygen.^2,^ ^3^

The classification of cerebral palsy is determined according to neuromuscular disorders and the distribution of disorders includes the spasticity (spastic type), the group with low muscle tension (hypotonic type), abnormal movement (dyskinetic type), stiffness (rigidity), balance disorder (ataxic type), and mixed disorder (mixed type).^4^ Other associated disorders such as seizures, mental retardation, eye and vision disorders, language and communication problems, hearing problems, and swallowing problems can also be found. Therefore, treatment requires the cooperation and coordination of the pediatric team, rehabilitation medicine, orthopedic surgeons, physical therapists, occupational therapists, therapists and psychologists to determine the appropriate treatment.

The spasticity type is the most common type of cerebral palsy^1^ that commonly affects gait from a low level, such as a decrease in walking speed or slight balance disorders, to a high level, such as having to use a wheelchair to move around all the time, and not being able to control the head or body to resist gravity. Treatment of this type of disorder ranges from the use of medications such as antispasmodic drugs, intramuscular injections, botulinum A toxin, intraspinal injections, and non-pharmacologic treatment such as physical therapy, splinting, and surgery.^5^ If the patient has mild symptoms, every patient should undergo physical therapy to make the muscles flexible without spasticity that will greatly affect the patient's walking, especially the hip muscles, thigh muscles, and leg muscles, as well as the upper limb muscles.^6^

However, because other provincial hospitals from the provincial to community level hospitals, the workload of the physiotherapy group is quite large, and Thailand has Thai traditional medicine as an alternative to treat various diseases. With reports that Thai massage can actually reduce muscle spasm in patients with cerebral ischemia, allowing patients to use the affected arm or leg well, and towards a better quality of life. In addition, Thai massage can also reduce stress and anxiety. Therefore, the application of traditional Thai massage to reduce muscle spasms to increase the efficiency of walking is another option that should be emphasized to reduce the burden on the physiotherapy group and increase the opportunity to access services for patients in areas where there are not enough physiotherapists.^8^

Nowadays, massage is increasingly used to treat diseases in medicine to reduce muscle spasms, such as

muscle spasms due to cerebral embolism (post stroke spasticity)^7^, multiple sclerosis^9^, cerebral palsy, etc. Most of them were found to be effective or equivalent to standard treatment.

Massage not only relaxes the patient, studies have shown that massage also has many benefits, such as helping to stretch stiff muscles, keeping muscle fibers elastic at the right level, breaking down the fascia that binds to the muscles, stimulating the circulatory system and the nervous supplied muscles to work better, etc.^7^ However, studies have shown that a small percentage of people who undergo massage experience side effects later, such as soreness, physical fatigue, headache, bruising, etc.

The effects of different types of massage, such as Swedish, Deep cross friction, Pakistani and Thai massage, on muscle stiffness in people with cerebral palsy compared to normal physical therapy.^11-15^ Table 1 showed that muscle stiffness is measured in the form of the Modified Ashworth Scale (MAS), abnormal reflex or Gross Motor Function Measure 88 (GMFM-88). It can be seen from past studies that there is still no There is a clear conclusion as to whether massage can actually reduce muscle spasms in patients with cerebral palsy, which may be due to a number of factors, such as the insufficient number of people participating in the study. The duration of the trial was not consistent with the treatment model. The format of the experiment was not a randomized controlled trial. Absence Concealment There is systematic bias, etc. (Table 2).

**Table 1** Massage Patterns and Duration ^11-15^


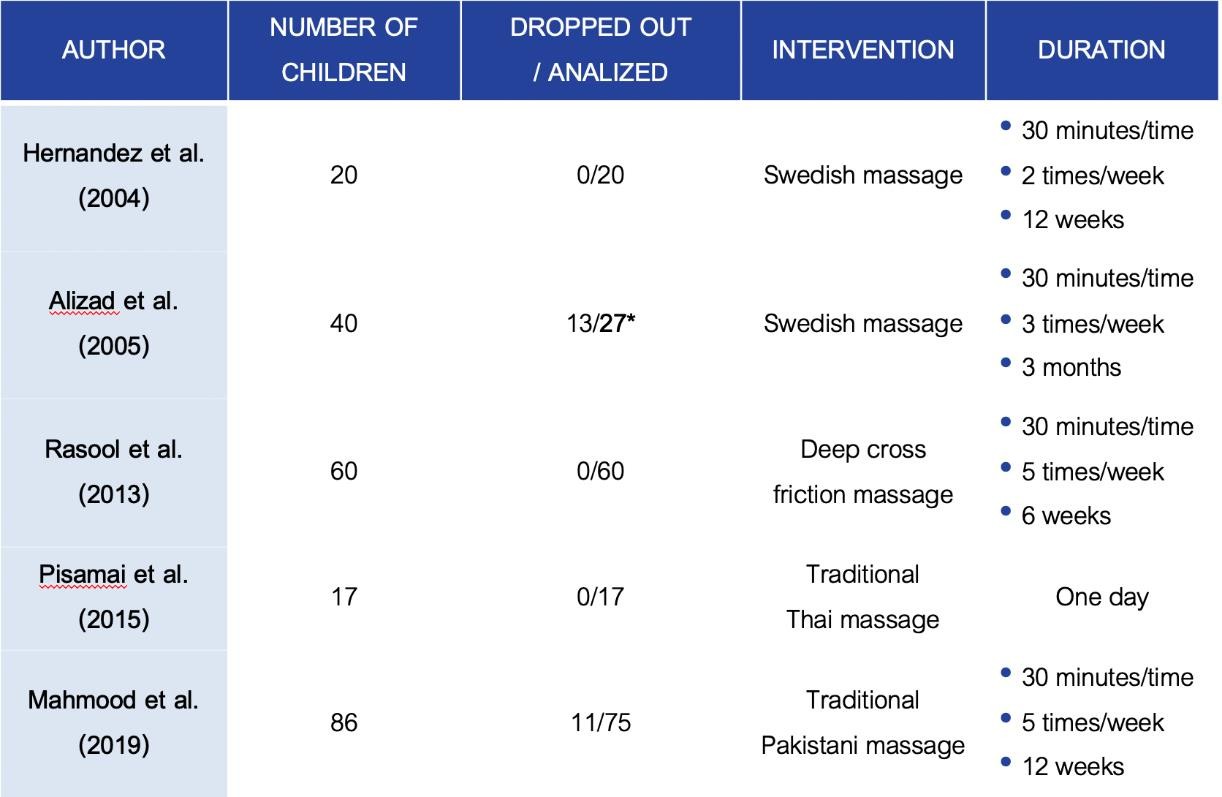


**Table 2** Research methodology and results of massage ^11-15^


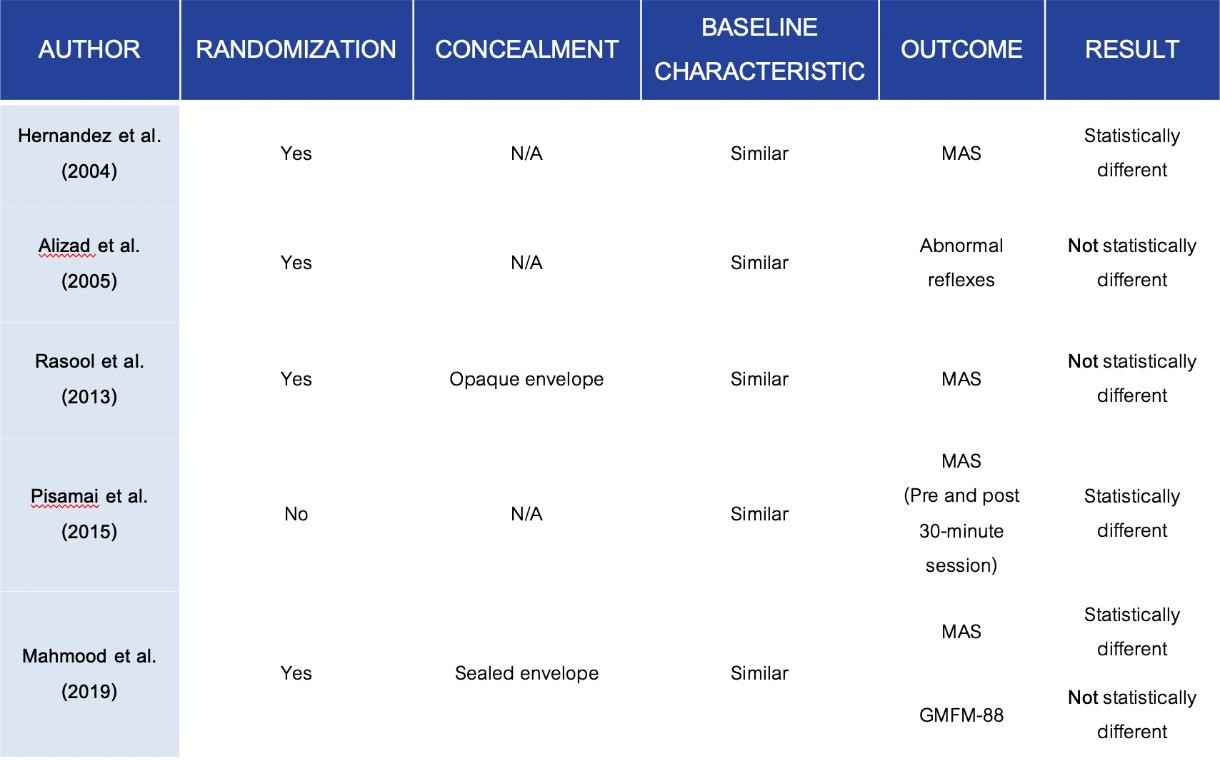


Reducing gait disorders caused by muscle spasm is another goal of treating the disease. Therefore, measuring the effect of massage on walking using a 3D gait analysis is another effective method. Because a large amount of quantitative data is collected during the test to increase the accuracy of analysis and evaluation of gait performance and reduce systematic error by displaying various forms of results such as temporal spatial, kinetics, kinematics, electromyography data, etc.^16^

This is because gait analyzers can provide large amounts of data, and they are quite complex. At present, representatives of such datasets have been invented for easy communication, such as the Gillette Gait Index (GGI), Gait Deviation Index (GDI), Gait Profile Score (GPS), etc^17^. The study found GPS represents the most useful dataset because no large reference dataset is required. GPS is calculated from 9 sets of Gait Variable Scores (GVS) combined to provide an overview of all walks. While GVS represents kinematics data comprises 9 sets of Pelvic tilt, Hip flexion/ extension, Knee flexion/ extension, Ankle dorsiflexion/ plantar flexion, Pelvic obliquity, Hip abduction / adduction, Pelvic rotation, Hip internal/ external rotation and Foot progression angle. GVS is calculated as the square root of the mean of the difference between the walker's kinematics data and the person's normal value (Averaged Normative Reference Dataset) squared, also known as Root Mean Square Error (RMSE). Therefore, the factors that affect GPS includes pelvic tilt and rotation, ability to flex/extend/rotate/adduct/abduct the hips, ability to flex/extend the knee, ability to dorsiflex/plantarflex the ankle. The higher GPS, the more gait abnomality.^17^

If there is precise processing, create a good research protocol to clearly define the duration of the experiment and the sample, it will lead to a very accurate description of the effect of massage on muscle tension that affects the gait of patients with cerebral palsy. Although the results did not differ between massage and physical therapy. If there were significant differences within the intervention group, Thai massage may also be another non-surgical treatment option and is considered to support Thai wisdom in the treatment of diseases.

# Objectives of the research project

To study and compare the effects of traditional Thai massage with regular physical therapy. In the development of gait in patients with spasticity, it was evaluated from the gait profile score.

- Primary outcome: Gait profile score (GPS)
- Secondary outcome: Modified Ashworth Scale, Temporal spatial data, Kinetics data, Oxygen consumption, Electromyography and Pedobarography

# Research Methods and Research Patterns Research Schedule

Research Model: Crossover randomized controlled trial

- Patients who meet the criteria and agree to participate in the research will be interviewed and examined to collect basic information and make an appointment for gait analysis.

- Participants will be analyzed by motion capture machine from Motion analysis corporation, Santa Rosa, CA, USA and recorded using Cortex 6.2 program. Data will be analyzed using Orthotrak and participants will be randomly assigned to group A and B.

- Participants will be taught home program physical therapy and will be taught from the beginning of the study to the end of the study for 18 weeks.

- Group A will receive traditional Thai massage according to the regulations mentioned above for 6 weeks, and then will be analyzed for a second time.

- Group B will receive regular physical therapy for 6 weeks, then will undergo a second walking analysis.

- Once Group A or B has undergone a second gait analysis, a 6-week interval will be given.

- Once the third gait analysis is undertaken, treatment will be alternated where group A will receive physical therapy as usual. Group B will receive a traditional Thai massage for 6 weeks.

- Both groups will then be analyzed for a fourth time.

- If the research participant is unable to come to Ramathibodi Hospital for physical therapy or Thai traditional massage every week because they live in a different province or are worried about the situation of communicable diseases such as COVID-19, etc., the research participant can use the treatment plan of the research conducted at Ramathibodi Hospital at the nearest hospital or can do it at home in order to facilitate the research participants in the current situation and prevent loss to follow-up treatment in the future (loss to follow-up) because it is a time-consuming research. The research team will teach the steps of physical therapy and Thai massage to the caregivers of the participants in detail. Video demonstration details of massage methods, and written procedures are created to ensure that caregivers are confident to do it by themself at home. In addition, the researcher will contact the participants every week to inquire problems found, verify accuracy and confirm that they have actually done it (Telemedicine). Moreover, there was research by Paul M Robinson et al.^22^ has compared physical therapy by a professional with physical therapy at home in patients with frozen shoulder after injecting water into the shoulder joint to destroy the fibrosis (hydrodilatation). It was found that the Oxford Shoulder Score (OSS) and EQ-5D were not significantly different.


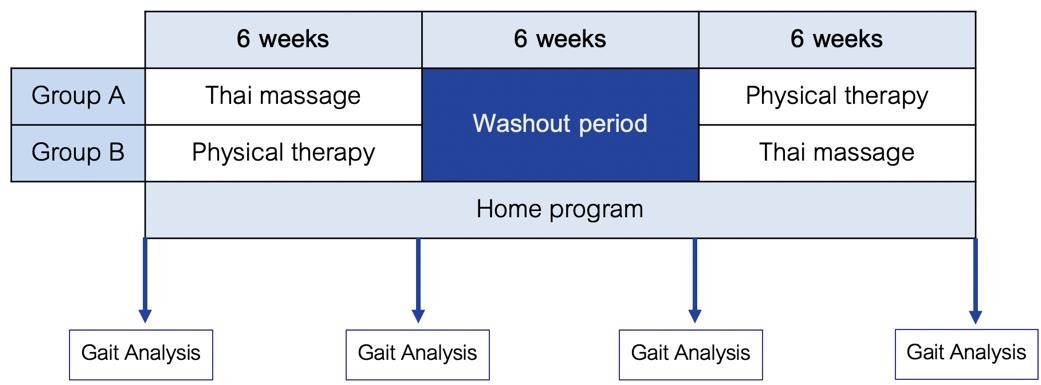


**Figure 3** Research Execution Chart

# Traditional Thai Massage

Two applied Thai medicine therapists with at least 3 years of experience (with a Thai traditional medical license) will provide the treatment, and undergo a diagnostic examination to assess both physical and emotional conditions before starting treatment. Massage is used by placing the thumb on the desired position and press the massage by adjusting the size and direction of the force applied to the weight. Appropriate according to the severity of the disease and age, it may be considered to stretch and relax the muscles with the paws according to the symptoms of the disease, softly, so that the recipient feels the most comfortable and will not press in a way that crushes, rub, or twists the muscles.

The basic massage will take about 10 seconds at each point, while the signal point massage will take about 30 seconds at each point, and the treatment will be placed in a supine, lateral decubitus, and sitting position as appropriate.

Traditional Thai Medicine Massage therapists will perform treatment according to the prescribed standards and procedures. The procedures for traditional Thai massage are adjusted according to participants, and Traditional Thai massage regulations for safety. Developing academic standard guidelines for massage have a total of 10 steps.

| \| 1. Massage the basic line of the legs, starting from the calves up to the thighs, and then back to the calves again. Then press the signal point on the front ankle and then press open the air door in the groin. \| 5 min \| \| --- \| --- \| \| 2. Massage the basic line of the back along the muscles from L5 to C7 level. \| 5 min \| \| 3. Massage the signal points of the outer legs, starting from the hip area, chasing along the thigh to the upper calf. Outer astragalus \| 5 min \| \| 4. Massage the signal points of the inner legs, starting from the thighs to the inner calves. \| 5 min \| \| 5. Massage the basic line of the inner arm, starting from the center of the inner upper arm to the front wrist. \| 2 min \| \| 6. Massage the basic line of the outer arm starting from the center of the outer upper arm to the center of the outer forearm. \| 2 min \| \| 7. Massage the basic shoulder line starting from the upper edge of the outer shoulder blade to the muscles around the nape of the neck, level C7. \| 2 min \| \| 8. Massage the basic line of the neck curve, starting from the nape of the neck, level C7, and chasing along the neck shaft to the base of the skull. \| 1 min \| \| 9. Massage signal points 1, 2 and 5 on the back of the head. At the base of the skull and massage the nerve point in the center of the crown. \| 2 min \| \| 10. Massage the 5 signal points in front of the head above the chin and above the lips, upper lip. \| 1 min \| |
| --- | --- | --- | --- | --- | --- | --- | --- | --- | --- | --- | --- | --- | --- | --- | --- | --- | --- | --- | --- | --- |

The massage will be carried out according to the aforementioned system for 2 days a week. 30 minutes

at a time for 6 consecutive weeks.

# Routine Physical Therapy

Physical therapy is popular in the treatment of patients with cerebral palsy. The purpose is to maintain the range of motion and muscle strength to maintain or improve it. Physical therapy can also help improve walking.

The physical therapy in this study was performed by a physical therapist with 5 years of experience (with a certificate in pathology) for a total of 6 weeks, 3 times a week for 40 minutes each time, for a total of 120 minutes/week, each time consisting of stretching exercise for 10 minutes, strengthening exercise for 15 minutes, and ambulatory training for 15 minutes. Stretching exercise and strength training will be selected according to specific affected muscle of each participant for safety e.g. gastrosoleus complex, tibialis posterior, peroneal muscle, rectus femoris, hamstring, hip adductor muscle, iliopsoas muscle, biceps brachaii.

# Home Program Physical Therapy

Participants or their parents will be taught physical therapy at home by doing it 5 times/week for at least 30 minutes each time, for a total of 150 minutes/week. The patient performs shoulder abduction/flexion, elbow flexion/extension, hip extension/abduction, and knee extension/flexion in repetitive movements for 3-5 minutes on both sides, and ambulatory training is the practice of moving both arms and legs forward, backward, left and right (side-to-side). Gait aid is allowed if available.

# Washout Period

This study set a washout period of 6 weeks, based on a study by Esin-Yi Kathy Cheng et al.^20^ that conducted a cross over randomized controlled trial using a 6-week washout period in children with cerebral palsy who were treated with whole body vibration versus physical therapy, and inferred from the literature that studied muscle spasms after a stroke. It was found that the patient began to have muscle stiffness at 6 weeks.^21^

By 6 weeks during the washout period, patients can still use walking aids/splints, plastics, or take their own medications as usual. However, they cannot receive other additional treatments (co-intervention) such as botulinum A toxin injections, physical therapy or any additional massage, additional surgery to correct muscle spasms, etc. There will be a follow-up phone call to inquire about the symptoms and whether they have received other treatments every 2 weeks. In case the temperature of the study participant is more than 38.0 degrees Celsius. Patients will not be able to undergo visual therapy, including Thai traditional massage. Patients can receive treatment in the next week or sooner. If the patient has a fever for more than 1 week and the cause cannot be determined, or the fever that requires a long treatment time, such as cancer, leukemia, malaria, pulmonary tuberculosis, etc., it will be necessary to withdraw from the research. However, the data of patients participating in the research will remain in the analysis.

Since the home program will start from the beginning of the first step analysis to the end of the experiment by the participants themselves or their parents will assist in doing it, during the washout period, the participants will be asked to do the home program like the period when they are treated with Thai massage or physical therapy according to the method specified above.

# Gait analysis

The steps of the gait analysis are as follows:

1. Wear a comfortable outfit to attach the movement measuring instrument and electromyography to the skin.

2. Attach a total of 29 motion capture devices according to Helen Hayes’ method.

3. Electromyography devices were attached using bipolar Ag/AgCl electrodes (3M Red dot, size 35 × 40 cm) on the skin at various locations including hip muscles, rectus femoris muscles, hamstring muscles, anterior tibial muscles, and posterior calf muscles.

4. Wear a device to measure oxygen consumption using the Oxycon mobile by CareFusion from Germany.

5. Measure the maximum voluntary contraction of each muscle group as mentioned above. To be used as the basis for individual analysis using ProEMG software, Myon 320 wireless EMG (Myon AG, Schwarzenberg Switzerland).

6. The participants were asked to walk in a straight line for about 8 meters for 10-15 laps with the same gait pattern. Measured by 3D motion capture and Coretex 6.2 software, data analysis with OrthoTRack 6.61 (Motion Analysis Corporation, Santa Rosa, CA, USA) and measurement of electromyography while walking. Select the 3 best rounds to calculate the kinematics and kinematics of the joints (in case it is not possible to attach both devices at the same time, each device will be separately installed and measured).

7. Participants were asked to stand and walk on a pedobarograph to measure their foot preference while standing and walking using the Sensor Medica Pedobarograph (Rome, Italy).


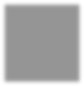

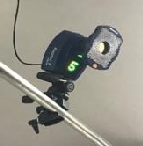

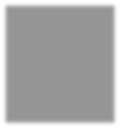

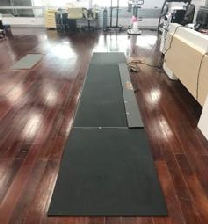

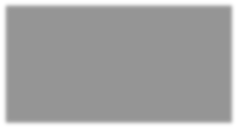

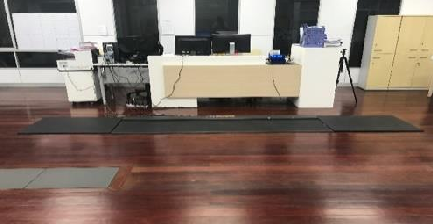

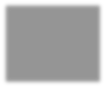

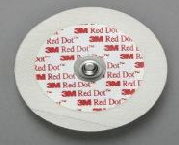

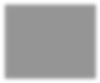

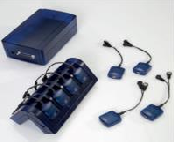


**Figure 3** 3D gait analysis equipment

| Research Schedule | Jul. 21 – Jul. 22 | Jul. 22 – Feb. 23 | Feb. 23 – May. 23 |
| --- | --- | --- | --- |
| Subject recruitment & operation |  |  |  |
| Data collection |  |  |  |
| Data analysis |  |  |  |

# Protocol Flow Chart

**
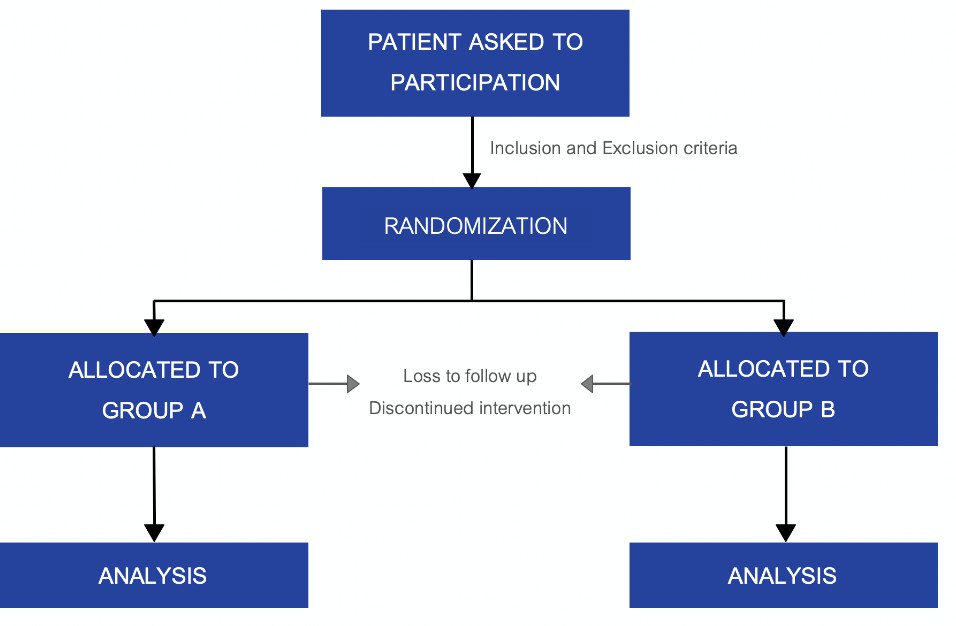
**

**Figure 4** CONSORT DIAGRAM

# Specify the number of research participants (Subject) to be studied, along with the entry and exclusion criteria.

Reference from mean GPS (Rasmussen HM, Nielsen DB, Pedersen NW, Overgaard S, Holsgaard-Larsen A. Gait Deviation Index, Gait Profile Score and Gait Variable Score in children with spastic cerebral palsy: Intra-rater reliability and agreement across two repeated sessions. Gait Posture. 2015; 42(2):133-7)

Use programmatic calculations STATA 16.0, Stata Corp, College Station, Texas, USA to estimate the sample size of difference two independent mean test due to a randomized controlled trial. Configure the variables as follows:

- Based on Alpha error 0.05 and power of the study 0.8

- Mean Gait Profile Score (GPS) of spastic cerebral palsy in the control group 13.90

- The difference in the mean Gait Profile Score (GPS) between the experimental group and the control group for the alternate hypothesis was 20 percent or 11.12.

- The standard deviation of the Gait Profile Score (GPS) of the control group was 4.9.

- Standard deviation of the Gait Profile Score (GPS) of the experimental group valued at 1.3

The sample size was calculated for each group of 26 cases.

# In case the sample size increases by 20 percent, the total sample size is 32 cases per group.

**Inclusion Criteria**

1. Patients with spastic cerebral palsy
2. Age over 5 years
3. Gross Motor Function Classification System (GMFCS) levels I to III (Figs. 2 and 3)
4. Agree to participate in the research project by signing

# Exclusion Criteria

1. Body temperature over 38.0 degrees Celsius.
2. Bleeding easily or difficult to stop
3. Fracture that has not yet been attached.
4. Fixed joint contracture
5. Skin infection.
6. Skin allergic rash. (contact dermatitis)
7. Patients treated with Botulinum A toxin injections within 6 months.
8. ADHD (attention deficit hyperactive disorder)
9. Behavioral disorders (behavioral disorder)
10. Uncontrolled epilepsy
11. Patients refuse or request to withdraw from the research project.

# Duration of the study (must begin after approval by the Human Research Ethics Committee)

After receiving the approval of ethics for human research for a period of 22 months.

# Expected risk or discomfort to the study participant.

Injuries during traditional Thai massage and physical therapy, such as myositis, bruises, or even walking accidents while participating in gait analysis, such as falls.

The research team has measures to prevent the occurrence of myositis or bruises from massage. Applied Thai medicine and physical therapists who have a license and work experience will provide treatment to the participants, and will assess muscle tension and provide appropriate treatment to each participant. In case of adverse events, the researcher will provide care according to the standard medical treatment to the best of their ability under the right to treatment of the research participants.

In regards with accidents during gait analysis, the research team will perform history taking and physical examination every time to assess whether the participants can walk continuously as required. If the participants need to use gait aids in their daily lives, they can use it while performing gait analysis as before. In addition, before every walking analysis, the environment in the room is checked for the risk of accidents, such as the slippery floor, obstruct walking items, room lighting, etc. If the above adverse events occur, the researcher will provide medical standard care to the best of his ability under the treatment rights of the research participants.

1. **Expected benefits** Obtain detailed gait information from the gait analyzer in order to exactly solve gait problems.

# Ethical considerations

Participants in the experiment will be described to every step of research protocol, the details of benefits and harms that may occur to the patient.

# Compensation to research participants (as necessary and appropriate) In the event of harm or unintended consequences to the participants of this study, what kind of care will the volunteers receive free of charge?

Participants will receive a stipend of 3,000 baht throughout the project. The researcher will be responsible for taking care of and preventing complications from the research. If such a situation occurs, the researcher will provide care according to the standard medical care to the best of their ability under the right to treatment of the research participants. Participants will receive standard treatment free of charge until the adverse effects of the study subside.

# In the case of receiving funding from the private sector, the details of the budget should be enumerated, and the name of the funder's coordinator and the phone number that can be contacted.

In the process of applying for a research grant from the Faculty of Medicine, Ramathibodi Hospital.

# Patient/Participant Information Sheet

- According to the attachment –

# Written consent with notice and willingness (Informed Consent Form)

- According to the attachment –

# Evidence or references

- 1. Graham HK, Thomason P, Novacheck TF. Cerebral Palsy. In: Stuart L. Weinstein M, John M. (Jack) Flynn M, editors. Lovell and Winter’s pediatric orthopedics. 1. 7th ed. Philadelphia2014. p. 484-549.
  2. MacLennan AH, Thompson SC, Gecz J. Cerebral palsy: causes, pathways, and the role of genetic variants. Am J Obstet Gynecol. 2015;213(6):779-88.
  3. Korzeniewski SJ, Slaughter J, Lenski M, Haak P, Paneth N. The complex aetiology of cerebral palsy. Nat Rev Neurol. 2018;14(9):528-43.
  4. Bax M, Goldstein M, Rosenbaum P, Leviton A, Paneth N, Dan B, et al. Proposed definition and classification of cerebral palsy, April 2005. Dev Med Child Neurol. 2005;47(8):571-6.
  5. Colver A, Fairhurst C, Pharoah PO. Cerebral palsy. Lancet. 2014;383(9924):1240-9.
  6. Franki I, Desloovere K, De Cat J, Feys H, Molenaers G, Calders P, et al. The evidence-base for basic physical therapy techniques targeting lower limb function in children with cerebral palsy: a systematic review using the International Classification of Functioning, Disability and Health as a conceptual framework. J Rehabil Med. 2012;44(5):385-95.
  7. Thanakiatpinyo T, Suwannatrai S, Suwannatrai U, Khumkaew P, Wiwattamongkol D, Vannabhum M, et al. The efficacy of traditional Thai massage in decreasing spasticity in elderly stroke patients. Clin Interv Aging. 2014;9:1311-9.
  8. Worachart Cheedchomchan. Dean of Physiotherapy 'Community Physical Therapist' Government shortage The reason for the system is not motivated. 2018 [cited 2020. Available from: [www.hfocus.org/content/2018/01/15261.](http://www.hfocus.org/content/2018/01/15261)
  9. Negahban H, Rezaie S, Goharpey S. Massage therapy and exercise therapy in patients with multiple sclerosis: a randomized controlled pilot study. Clin Rehabil. 2013;27(12):1126-36.
  10. Cambron JA, Dexheimer J, Coe P, Swenson R. Side Effects of Massage Therapy; a Pilot Study. National University of Health Sciences.
  11. Alizad V, Vameghi R, Sajedi F, Alaeddini F, Jazy MRH. Swedish massage and abnormal reflexes of children with spastic cerebral palsy. Iranian Rehabilitation Journal. 2007;5(5,6):30-3.
  12. Rasool F, Memon AR, Kiyani MM, Sajjad AG. The effect of deep cross friction massage on spasticity of children with cerebral palsy: A double-blind randomised controlled trial. J Pak Med Assoc. 2017;67(1):87-91.
  13. Malila P, Seeda K, Machom S, Eungpinithpong W. Effects of Thai Massage on Spasticity in Young People with Cerebral Palsy. J Med Assoc Thai. 2015;98 Suppl 5:S92-6.
  14. Mahmood Q, Habibullah S, Babur MN. Potential effects of traditional massage on spasticity and gross motor function in children with spastic cerebral palsy: A randomized controlled trial. Pak J Med Sci. 2019;35(5):1210-5.
  15. Hernandez-Reif M, Feild T, Largie S, Diego M, Manigat N, Seoanes J, et al. Cerebral palsy symptoms in children decreased following massage therapy. Early Child development and Care. 2005;175(5):445-56.
  16. Armand S, Decoulon G, Bonnefoy-Mazure A. Gait analysis in children with cerebral palsy. EFORT open reviews. 2016;1:448-58.
  17. Holmes SJ, Mudge AJ, Wojciechowski EA, Axt MW, Burns J. Impact of multilevel joint contractures of the hips, knees and ankles on the Gait Profile score in children with cerebral palsy. Clin Biomech (Bristol, Avon). 2018;59:8-14.
  18. Baker R, McGinley JL, Schwartz MH, Beynon S, Rozumalski A, Graham HK, et al. The gait profile score and movement analysis profile. Gait Posture. 2009;30(3):265-9.
  19. Apichart Limtiyayothin, Somporn Nongbuadee. Introduction to Thai Massage in the Royal Court. Ayurvedic School, Applied Thai Medicine Institute, Faculty of Medicine, Siriraj Hospital. Mahidol University, editors. Hand Medicine Thai Traditional Karma (Royal Thai Massage), Part 1: Basic Massage.
  20. Cheng HY, Yu YC, Wong AM, Tsai YS, Ju YY. Effects of an eight-week whole body vibration on lower extremity muscle tone and function in children with cerebral palsy. Res Dev Disabil. 2015;38:256-61.
  21. Thibaut A, Chatelle C, Ziegler E, Bruno MA, Laureys S, Gosseries O. Spasticity after stroke: physiology, assessment and treatment. Brain Inj. 2013;27(10):
  22. Robinson PM, Norris J, Roberts CP. Randomized controlled trial of supervised physiotherapy versus a home exercise program after hydrodilatation for the management of primary frozen shoulder. J Shoulder Elbow Surg. 2017 May;26(5):757-765. doi: 10.1016/j.jse.2017.01.012. Epub 2017 Mar 18. PMID: 28318848.

**Appendix**

**Data Record Form**

| **Part 1** : Preliminary Participant Information | | For staff to fill in only. |
| --- | --- | --- |
| 1.1  1.2  1.3  1.4  1.5  1.6  1.7  1.8  1.9  1.10  1.11  1.12  1.13 | Date of participation in the study: ………/………../………  Name: ……………..…………………………………………………...  Sex ❒ 1. male ❒ 2. female  Age: ………. year  Weight: …………. kilogram  Height: ………….. centimetre  Muscle spasm pattern:  ❒ 1. spastic diplegia  ❒ 2. spastic hemiplegia  ❒ 3. spastic quadriplegia  Gross Motor Function Classification System   (GMFCS) level  ❒ 1. I ❒ 2. II ❒ 3. III ❒ 4. IV  Birth weight:  ❒ 1. < 1000 gram  ❒ 2. 1000 – 1499 gram  ❒ 3. 1500 – 2500 gram  ❒ 4. > 2500 gram  Birth pattern: ❒ 1.Natural birth ❒ 2.C-section  Birthplace:  ❒ 1. Government Hospital ❒ 2. Private hospitals  Complications during childbirth/postpartum:  …………………………………………………………………  Treatment before participating in the study and year:  ………………………………………………………………… | Date [ ][ ] / [ ][ ] / [ ][ ]  Name [ ]  Sex [ ]  Age [ ]  Weight [ ] [ ] [ ]  Height [ ] [ ] [ ]  Spastic type [ ]  GMFCS [ ]  Birth weight [ ]  Mode of delivery [ ]  Birth place [ ]  Complication [ ]  Previous treatment [ ] |

**Part 2:** Physical Examination

2.1 Mobility scales

|  | | Passive ROM | | Active ROM | | Muscle Power | |
| --- | --- | --- | --- | --- | --- | --- | --- |
|  |  | Right | Left | Right | Left | Right | Left |
| 2.1.1 | Hip Flexion |  |  |  |  |  |  |
| 2.1.2 | Hip Extension |  |  |  |  |  |  |
| 2.1.3 | Hip Abduction |  |  |  |  |  |  |
| 2.1.4 | Hip Adduction |  |  |  |  |  |  |
| 2.1.5 | Hip Internal Rotation |  |  |  |  |  |  |
| 2.1.6 | Hip External Rotation |  |  |  |  |  |  |
| 2.1.7 | Knee Flexion |  |  |  |  |  |  |
| 2.1.8 | Knee Extension |  |  |  |  |  |  |
| 2.1.8 | Popliteal Angle |  |  | - | - | - | - |
| 2.1.9 | Ely’s test |  |  | - | - | - | - |
| 2.1.10 | Ankle Dorsiflexion with knee extension |  |  |  |  |  |  |
| 2.1.11 | Ankle Dorsiflexion with knee flexion |  |  |  |  |  |  |
| 2.1.12 | Ankle Plantar flexion |  |  |  |  |  |  |
| 2.1.13 | Ankle Inversion |  |  |  |  |  |  |
| 2.1.14 | Ankle Eversion |  |  |  |  |  |  |

2.2 Motor control

| Muscle | | Right | Left | **Good**: Patient is able to isolate individual muscle contraction through entire available passive ROM upon command.  **Fair**: Patient is able to initiate muscle contraction upon command, but it unable to completely isolate contraction through entire passive ROM.  **Poor**: Patient is unable to isolate individual muscle contraction through entire available passive ROM secondary to synergistic patterns, increase tone and/or decrease or absent activation. |
| --- | --- | --- | --- | --- |
| 2.2.1 | Iliopsoas |  |  |  |
| 2.2.2 | Gluteus Maximus |  |  |  |
| 2.2.3 | Quadriceps |  |  |  |
| 2.2.4 | Hamstring |  |  |  |
| 2.2.5 | Anterior Tibialis |  |  |  |
| 2.2.6 | Gastrocnemius |  |  |  |
| 2.2.7 | Posterior Tibialis |  |  |  |
| 2.2.8 | Soleus |  |  |  |
| 2.2.9 | Peroneus |  |  |  |

2.3 Muscle tone

| Muscle | | Right | Left | **Modified Ashworth scale**  0: No increase in tone  1: Slight increase in muscle tone, manifested by a catch and release or minimal resistance at  the end of the ROM when the affected part(s) is moved in flexion or extension  1+: Slight increase in muscle tone, manifested by a catch, followed by minimal resistance  throughout the remainder (less than half) of the ROM  2: More marked increase in muscle tone through most of the ROM, but affected part(s) easily  moved  3: Considerable increase in muscle tone, passive movement difficult  4: Affected part(s) rigid in flexion or extension |
| --- | --- | --- | --- | --- |
| 2.3.1 | Iliopsoas |  |  |  |
| 2.3.2 | Gluteus Maximus |  |  |  |
| 2.3.3 | Adductor |  |  |  |
| 2.3.4 | Rectus Femoris |  |  |  |
| 2.3.5 | Hamstring |  |  |  |
| 2.3.6 | Anterior Tibialis |  |  |  |
| 2.3.7 | Gastrocnemius |  |  |  |
| 2.3.8 | Posterior Tibialis |  |  |  |
| 2.3.9 | Soleus |  |  |  |
| 2.3.10 | Peroneus |  |  |  |
| 2.3.11 | Clonus |  |  |  |

**Part 3:** 3D gait analysis

- 1. Temporal spatial

|  | | | **Subject** | **St.Dev** | **Norm** | **St.Dev** | **%Norm** |
| --- | --- | --- | --- | --- | --- | --- | --- |
| 3.1.1 | Velocity | (cm/s) |  |  |  |  |  |
| 3.1.2 | Cadence | (steps/min) |  |  |  |  |  |
| 3.1.3 | Stride Length | (cm) |  |  |  |  |  |
| 3.1.4 | Step Width | (cm) |  |  |  |  |  |
| 3.1.5 | Pelvic Width | (cm) |  |  |  |  |  |
| 3.1.6 | Pelvic to Step Ratio | (cm) |  |  |  |  |  |
|  |  |  |  |  |  |  |  |
|  |  |  |  |  |  |  |  |
| **Right** | | | **Subject** | **St.Dev** | **Norm** | **St.Dev** | **%Norm** |
| 3.1.7 | Step Length | (cm) |  |  |  |  |  |
| 3.1.8 | Weight Accept | (% cycle) |  |  |  |  |  |
| 3.1.9 | Single Support | (% cycle) |  |  |  |  |  |
| 3.1.10 | Weight Release | (% cycle) |  |  |  |  |  |
| 3.1.11 | Stance | (% cycle) |  |  |  |  |  |
| 3.1.12 | Swing | (% cycle) |  |  |  |  |  |
|  |  |  |  |  |  |  |  |
|  |  |  |  |  |  |  |  |
| **Left** | | | **Subject** | **St.Dev** | **Norm** | **St.Dev** | **%Norm** |
| 3.1.13 | Step Length | (cm) |  |  |  |  |  |
| 3.1.14 | Weight Accept | (% cycle) |  |  |  |  |  |
| 3.1.15 | Single Support | (% cycle) |  |  |  |  |  |
| 3.1.16 | Weight Release | (% cycle) |  |  |  |  |  |
| 3.1.17 | Stance | (% cycle) |  |  |  |  |  |
| 3.1.18 | Swing | (% cycle) |  |  |  |  |  |

- The patient walked ………. cm/sec with cadence at ………. steps/min.
- Stride length was ………. cm. and step width was ………. cm.
- Right step length was ………. cm. and the left was ………. cm.
- Stance/Swing phase was ……….% and ……….% at the right limb; ……….% and ……….% at the left side, respectively

3.2 Kinematic

**Right lower extremity kinematic**

…………………….……………………………………………………………………………………………………………………………………………………………………………………………………………………………………………………………………………………………………………………………………………………………………………………………………………………………………………………………………………………………………………………………………………………………………………………………………………………………………………………………………………………………………………………………………………………………………………………………………………………………………………………………………………………………………………………………………………………………………………………………………………………………………………………………………………………………………………………………………………………………………………………………………………………………………………………………………………………………………………………………………………………………………………………………………………………………………………………………………………………

**Left lower extremity kinematic**

…………………….……………………………………………………………………………………………………………………………………………………………………………………………………………………………………………………………………………………………………………………………………………………………………………………………………………………………………………………………………………………………………………………………………………………………………………………………………………………………………………………………………………………………………………………………………………………………………………………………………………………………………………………………………………………………………………………………………………………………………………………………………………………………………………………………………………………………………………………………………………………………………………………………………………………………………………………………………………………………………………………………………………………………………………………………………………………………………………………………………………………

3.3 Kinetic

**Right kinetic**

…………………….……………………………………………………………………………………………………………………………………………………………………………………………………………………………………………………………………………………………………………………………………………………………………………………………………………………………………………………………………………………………………………………………………………………………………………………………………………………………………………………………………………………………………………………………………………………………………………………………………………………………………………………………………………………………………………………………………………………………………………………………………………………………………………………………………………………………………………………………………………………………………………………………………………………………………………………………………………………………………………………………………………………………………………………………………………………………………………………………………………………

**Left kinetic**

…………………….……………………………………………………………………………………………………………………………………………………………………………………………………………………………………………………………………………………………………………………………………………………………………………………………………………………………………………………………………………………………………………………………………………………………………………………………………………………………………………………………………………………………………………………………………………………………………………………………………………………………………………………………………………………………………………………………………………………………………………………………………………………………………………………………………………………………………………………………………………………………………………………………………………………………………………………………………………………………………………………………………………………………………………………………………………………………………………………………………………………

3.4 Trunk and Pelvis

…………………….…………………………………………………………………………………………………………………………………………………………………………………………………………………………………………………………………………………………………………………………………………………………………………………………………………………………………………………………………………………………………………………………………………

3.5 Muscle activity

…………………….……………………………………………………………………………………………………………………………………………………………………………………………………………………………………………………………………………………………………………………………………………………………………………………………………………………………………………………………………………………………………………………………………………………………………………………………………………………………………………………………………………………………………………………………………………………………………………………………………………………………………………………………………………………………………………………………………………………………………………………………………………………………………………………………………………………………………………………………………………………………………………………………………………………………………………………………………………………………………………………………………………………………………………………………………………………………………………………………………………………

3.6 Oxygen consumption

…………………….……………………………………………………………………………………………………………………………………………………………………………………………………………………………………………………………………………………………………………………………………………………………………………………………………………………………………………………………………………………………………………………………………………………………………………………………………………………………………………………………………………………………………………………………………………………………………………………………………………………………………………………………………………………………………………………………………………………………………………………………………………………………………………………………………………………………………………………………………………………………………………………………………………………………………………………………………………………………………………………………………………………………………………………………………………………………………………………………………………………
