# Supplementary material for: The effect of traditional Thai massage vs routine physical therapy on gait pattern in spastic cerebral palsy: A cross-over randomized controlled trial
Source: PLoS One. 2025 May 29;20(5):e0325169. doi: 10.1371/journal.pone.0325169 (PMC12122028; doi:10.1371/journal.pone.0325169)
Supplement: S2 Protocol — (DOCX) [file pone.0325169.s003.docx]

# แบบเสนอโครงการวิจัยเพื่อรับการพิจารณาจากคณะกรรมการจริยธรรมการวิจัยในคน คณะแพทยศาสตร์โรงพยาบาลรามาธิบดี มหาวิทยาลัยมหิดล (ฉบับเต็ม)

1. **ชื่อโครงการ**

การศึกษาเปรียบเทียบผลของการนวดแผนไทยโบราณกับการทำกายภาพบำบัดต่อรูปแบบการเดินของผู้ป่วย

สมองพิการ

# ชื่อหัวหน้าโครงการวิจัย

นายแพทย์พีรพัฒน์ เลิศวิราม

# ชื่อผู้ร่วมการวิจัย

ศาสตราจารย์ ดร. แพทย์หญิงภัทรวัณย์ วรธนารัตน์

อ.พญ.อภิพรรณ เอี่ยมชัยมงคล\

พท.ป.สุชานนท์ เบ้าสุวรรณ

พท.ป.พนธ์ศภัส องค์ธนะสิน

กภ.พิมพ์พิสุทธ์ ศรีนรสิทธ์

ภ.กรองแก้ว แกล้วกสิกรรม

กภ.ธันยพร ปะทะธง

# บทนำ หลักการและเหตุผล

ภาวะสมองพิการ (Cerebral palsy) คือ กลุ่มอาการที่มีความผิดปกติของร่างกายในส่วนของท่าทางและการ เคลื่อนไหว ซึ่งเกิดจากพยาธิสภาพในสมองที่คงที่ ซึ่งเกิดในช่วงที่สมองกำลังเจริญเติบโตและพัฒนาอยู่ โดยถือเป็น ความพิการที่พบบ่อยในกลุ่มประเทศที่พัฒนาแล้ว โดยสามารถพบได้ถึง 7 คนต่อการเกิด 1000 คน^1^

สาเหตุของภาวะสมองพิการสามารถเกิดได้ทั้งในช่วงระหว่างการตั้งครรภ์ เช่น ภาวะความดันโลหิตสูง การติด เชื้อ การได้รับสารพิษ โรคหัวใจและการหายใจ รวมถึงโรคทางเมทาบอลิซึม เป็นต้น ช่วงระหว่างการคลอด เช่น รก เกาะต่ำ ช่วงเวลาการคลอดนานกว่าปกติ ภาวะสายสะดือถูกกดทับ ท่าคลอดที่ผิดปกติ ขนาดทารกไม่สัมพันธ์กับเชิง กรานมารดา ภาวะคลอดก่อนกำหนด เป็นต้น และช่วงหลังคลอด เช่น การบาดเจ็บของศีรษะ ความผิดปกติของเส้น เลือดในสมอง ความเจ็บป่วยที่ก่อให้เกิดสมองขาดเลือดหรือออกซิเจน^2,^ ^3^

การแบ่งชนิดของภาวะสมองพิการจะพิจารณาตามความผิดปกติของระบบประสาทและกล้ามเนื้อ และการ กระจายตัวของความผิดปกติ ได้แก่ กลุ่มที่มีอาการเกร็ง (spastic type) กลุ่มที่มีความตึงตัวของกล้ามเนื้อน้อย (hypotonic type) กลุ่มที่มีความผิดปกติของการเคลื่อนไหว (dyskinetic type) กลุ่มที่มีอาการแข็งเกร็ง (rigidity) กลุ่มที่มีปัญหาการทรงตัว (ataxic type) และกลุ่มผสม (mixed type)^4^ นอกจากนี้ยังสามารถพบความผิดปกติอื่น ร่วมได้ เช่น อาการชัก ภาวะปัญญาอ่อน ความผิดปกติของตาและการมองเห็น ปัญหาด้านภาษาและการสื่อสาร ปัญหาการได้ยินและปัญหาการดูดกลืน จะเห็นได้ว่าภาวะสมองพิการส่งผลต่อระบบการทำงานของร่างกายหลาย ระบบและการใช้ชีวิตประจำวัน ดังนั้นการรักษาจึงต้องอาศัยความร่วมและประงานสานกันของทีมกุมารแพทย์ แพทย์เวชศาสตร์ฟื้นฟู แพทย์ศัลยกรรมกระดูกและข้อ ทีมนักกายภาพบำบัด ทีมนักกิจกรรมบำบัด นักอรรถบำบัด และนักจิตวิทยา เพื่อพิจารณาการรักษาที่เหมาะสม

กลุ่มที่มีอาการเกร็ง (spastic type) เป็นกลุ่มที่พบบ่อยที่สุดในภาวะสมองพิการ^1^ มักส่งผลต่อการเดินตั้งแต่ใน ระดับต่ำ เช่น ความเร็วในการเดินลดลง หรือการทรงตัวผิดปกติเล็กน้อย ไปจนถึงระดับสูง เช่น ต้องใช้รถเข็นใน การเคลื่อนย้ายตลอดเวลาและไม่สามารถควบคุมศีรษะหรือลำตัวเพื่อต้านแรงโน้มถ่วงได้ การรักษาของความ ผิดปกติชนิดนี้มีตั้งแต่การใช้ยา เช่น ยาลดการแข็งเกร็งของกล้ามเนื้อ การฉีดยาเข้ากล้ามเนื้อ การใช้ botulinum A toxin การฉีดยาเข้าเยื่อหุ้มไขสันหลัง และแบบไม่ใช้ยา เช่น การทำกายภาพบำบัด การใส่เฝือก และการผ่าตัด^5^ หาก ผู้ป่วยมีอาการไม่มาก ผู้ป่วยควรได้รับการทำกายภาพบำบัดทุกรายเพื่อทำให้กล้ามเนื้อมีความยืดหยุ่น ไม่ให้เกิด ภาวะกล้ามเนื้อแข็งเกร็ง ซึ่งจะส่งผลต่อการเดินของผู้ป่วยเป็นอย่างมาก โดยเฉพาะกล้ามเนื้อสะโพก กล้ามเนื้อต้นขา และกล้ามเนื้อขา รวมถึงกล้ามเนื้อรยางค์บน^6^

อย่างไรก็ตามเนื่องจากโรงพยาบาลในต่างจังหวัดตั้งแต่ในระดับโรงพยาบาลจังหวัดไปถึงโรงพยาบาลชุมชน ภาระงานของกลุ่มงานกายภาพบำบัดมีค่อนข้างมาก และประเทศไทยมีแพทย์แผนไทยประยุกต์เป็นอีกทางเลือก หนึ่งในการรักษาโรคต่าง ๆ โดยมีการรายงานว่าการนวดแผนไทยสามารถลดการเกร็งของกล้ามเนื้อได้จริงในกลุ่ม ผู้ป่วยสมองขาดเลือด ทำให้ผู้ป่วยสามารถใช้งานแขนหรือขาข้างที่ได้รับผลกระทบได้ดี และนำไปสู่คุณภาพชีวิตที่ดี ยิ่งขึ้น นอกจากนี้การนวดแผนไทยยังสามารถลดความเครียดและวิตกกังวลได้อีกด้วย โดยผ่านการกระตุ้นระบบ ประสาท parasympathetic^7^ ดังนั้นการประยุกต์ใช้การนวดแผนไทยโบราณมาใช้ในการลดอาการเกร็งของกล้ามเนื้อ เพื่อเพิ่มประสิทธิภาพในการเดินก็เป็นอีกทางเลือกที่ควรให้ความสำคัญ เพื่อลดภาระของกลุ่มงานกายภาพบำบัด และเพิ่มโอกาสในการเข้าถึงการบริการของผู้ป่วยในพื้นที่ที่ไม่มีนักกายภาพบำบัดเพียงพอ^8^

ในปัจจุบันการนวดถูกนำมาใช้รักษาโรคในทางการแพทย์มากขึ้นเพื่อลดอาการเกร็งของกล้ามเนื้อ เช่น

ภาวะกล้ามเนื้อเกร็งจากภาวะหลอดเลือดสมองอุดตัน (post stroke spasticity)^7^, โรคเอ็มเอส (multiple sclerosis)^9^, ภาวะสมองพิการ (cerebral palsy) เป็นต้น โดยพบว่าส่วนใหญ่ได้ผลดี หรือเทียบเท่ากับการรักษาแบบมาตรฐาน

การนวดนอกจากจะทำให้ผู้ป่วยเกิดความผ่อนคลายแล้ว จากการศึกษาพบว่าการนวดยังมีประโยชน์อีก มากมาย เช่น สามารถช่วยยืดกล้ามเนื้อที่แข็งเกร็งได้ ช่วยทำให้เส้นใยกล้ามเนื้อมีความยืดหยุ่นในระดับเหมาะสม ช่วยสลายผังผืดที่ยึดเกาะกล้ามเนื้อให้สลายไปได้ ช่วยกระตุ้นระบบไหวเวียนเลือดและระบบประสาทที่ไปเลี้ยง กล้ามเนื้อให้ทำงานได้ดีขึ้น เป็นต้น^7^ อย่างไรก็ตามจากการศึกษาพบว่ามีผู้เข้ารับการนวดส่วนน้อยเกิดผลข้างเคียง ภายหลัง เช่น อาการปวดระบม (soreness) ร่างกายอ่อนเพลีย (fatigue) ปวดศีรษะ เกิดจ้ำเลือดตามตัว (bruising) เป็น ต้น^10^

จากการทบทวนวรรณกรรมพบว่ามีการศึกษาผลของการนวดในแบบต่าง ๆ เช่น การนวดแบบ Swedish, Deep cross friction, Pakistani และ Thai massage ต่อการเกร็งของกล้ามเนื้อในกลุ่มผู้ป่วยภาวะสมองพิการโดยเทียบ กับการทำกายภาพแบบปกติ^11-15^ ดังตารางที่ 1 โดยความแข็งเกร็งของกล้ามเนื้อถูกวัดในรูปแบบของ Modified Ashworth Scale (MAS), abnormal reflex หรือ Gross Motor Function Measure 88 (GMFM-88) ซึ่งมีบางงานวิจัย ที่สนับสนุนว่าการนวดนั้นสามารถทำให้ระดับการเกร็งของกล้ามเนื้อลดลงอย่างมีนัยสำคัญ เมื่อเทียบกับการทำ กายภาพบำบัด^13-15^ แต่ก็ยังมีงานวิจัยที่ไม่สนับสนุนผลลัพธ์ข้างต้น^11,^ ^12,^ ^14^ ซึ่งเห็นได้ว่าจากการศึกษาที่ผ่านมายังไม่ เกิดข้อสรุปที่ชัดเจนว่าการนวดสามารถลดอาการเกร็งของกล้ามเนื้อในผู้ป่วยสมองพิการได้จริงหรือไม่ ซึ่งอาจเกิด จากปัจจัยหลายอย่าง เช่น จำนวนบุคคลเข้าร่วมงานวิจัยที่ไม่เพียงพอ การกำหนดระยะเวลาในการทดลองไม่ สอดคล้องต่อรูปแบบการรักษา รูปแบบการทดลองไม่ใช่การทดลองแบบสุ่ม (randomized controlled trial) การไม่มี การปกปิด (concealment) มีความผิดพลาดเชิงระบบ (systematic bias) เป็นต้น (ตารางที่ 2)

**ตารางที่ 1** รูปแบบและระยะเวลาการนวด ^11-15^


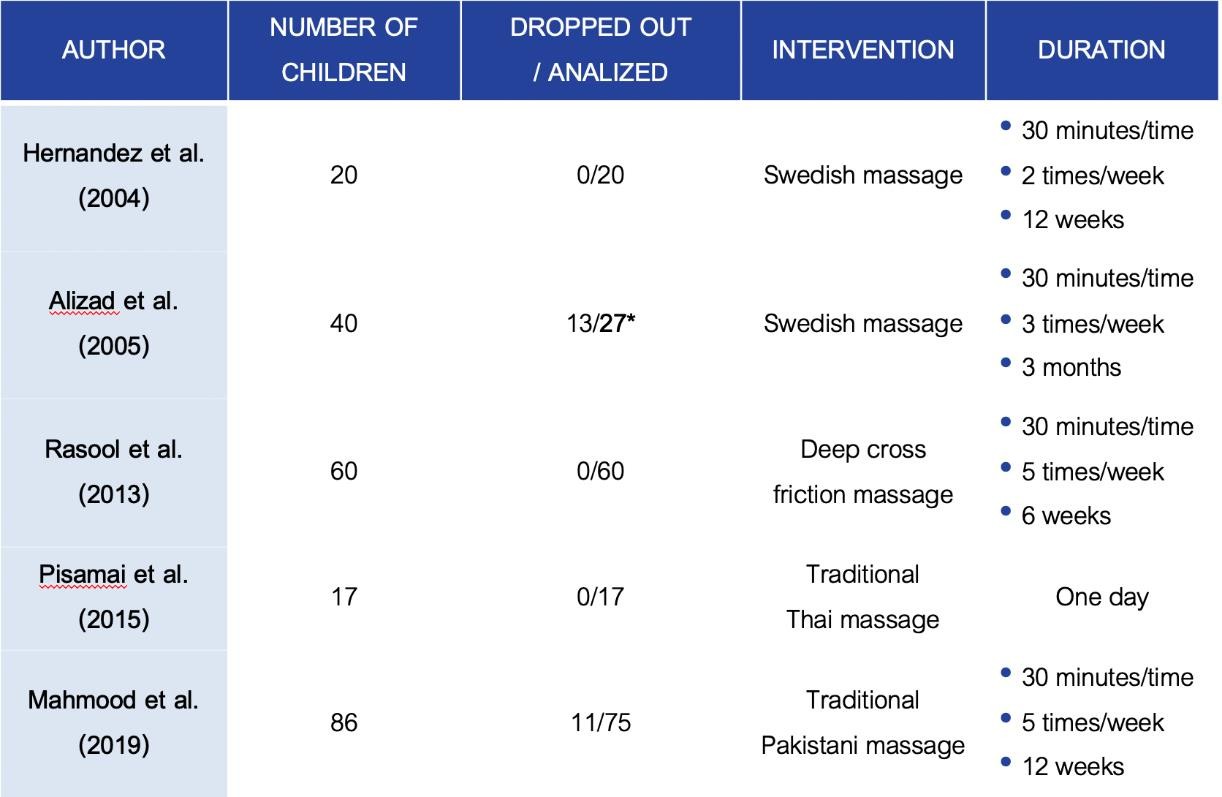


**ตารางที่ 2** ระเบียบวิธีวิจัยและผลของการนวด ^11-15^


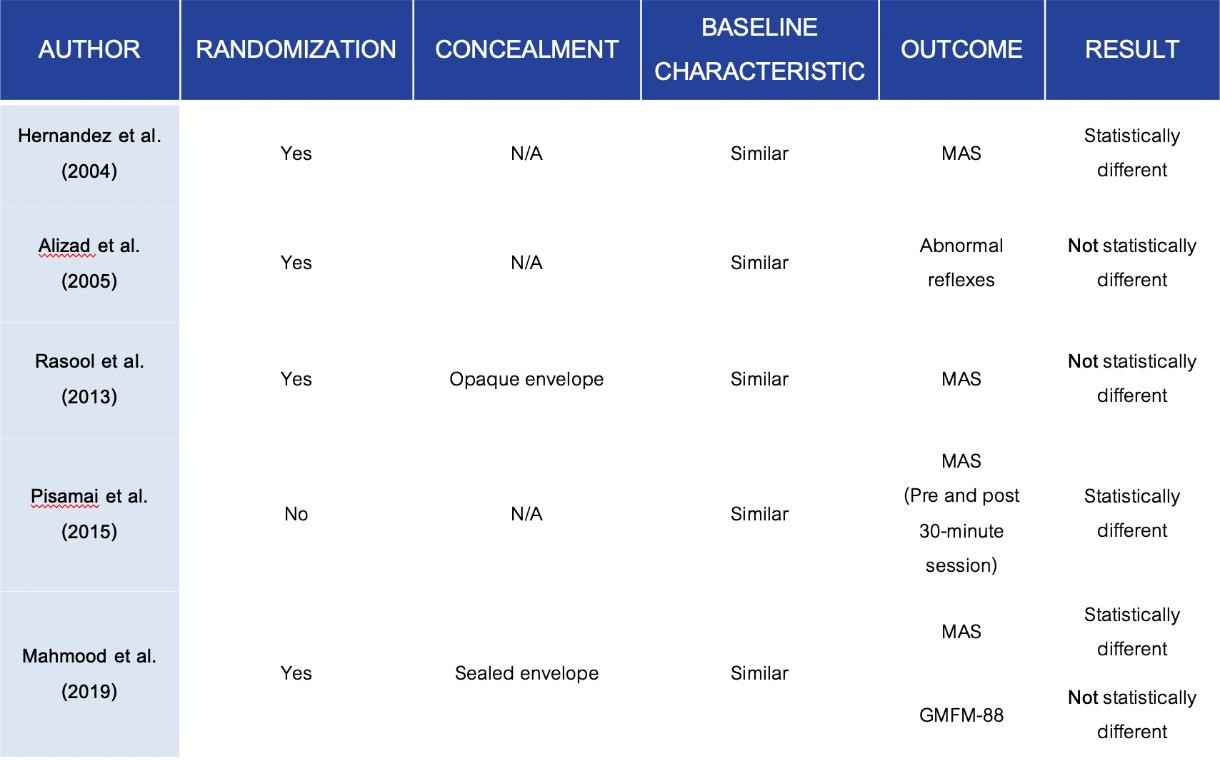


การลดความผิดปกติของการเดินที่เกิดจากการเกร็งของกล้ามเนื้อ เป็นอีกหนึ่งเป้าหมายของการรักษาภาวะ สมองพิการ ดังนั้นการวัดผลของการนวดต่อการเดินโดยใช้เครื่องวิเคราะห์การเดินรูปแบบสามมิติ (3D gait analysis) จึงเป็นอีกหนึ่งวิธีที่มีประสิทธิภาพ เนื่องจากมีการเก็บข้อมูลเชิงปริมาณในปริมาณมากในขณะทดสอบ เพื่อเพิ่มความแม่นยำในการวิเคราะห์และประเมินประสิทธิภาพของการเดิน และลดความผิดพลาดเชิงระบบ (systematic error) โดยแสดงผลออกมาหลากหลายรูปแบบ ได้แก่ temporal spatial, kinetics, kinematics, electromyography data เป็นต้น^16^

เนื่องจากเครื่องวิเคราะห์การเดินสามารถให้ข้อมูลปริมาณมากและค่อนข้างซับซ้อน ในปัจจุบันจึงมีการ คิดค้นตัวแทนของชุดข้อมูลดังกล่าวเพื่อง่ายต่อการสื่อสาร เช่น Gillette Gait Index (GGI), Gait Deviation Index (GDI), Gait Profile Score (GPS) เป็นต้น^17^ และจากการศึกษาพบว่าการใช้ Gait Profile Score (GPS) เป็นตัวแทนชุดข้อมูลมีความสะดวกต่อการใช้งานมากที่สุด เนื่องจากไม่จำเป็นต้องใช้ฐานข้อมูลอ้างอิงใน ปริมาณมากในการคำนวณ (large reference dataset)^18^ โดย Gait Profile Score(GPS) คำนวณจาก Gait Variable Score (GVS) 9 ชุดมารวมกันเพื่อแสดงภาพรวมของการเดินทั้งหมด ในขณะที่ Gait Variable Score (GVS) เป็นตัวแทนของ kinematics data มีทั้งหมด 9 ชุด ไ ด้แก่ Pelvic tilt, Hip flexion/ extension, Knee flexion/ extension, Ankle dorsiflexion/ plantar flexion, Pelvic obliquity, Hip abduction / adduction, Pelvic rotation, Hip internal/ external rotation และ Foot progression angle ซึ่งสามารถบอกความผิดปกติของการเดินเป็นตำแหน่งได้ โดย Gait Variable Score (GVS) คำนวณจากรากที่สองของค่าเฉลี่ยของผลต่างระหว่าง kinematics data ของผู้เดิน กับค่าปกติของบุคคลทั่วไป (averaged normative reference dataset) ยกกำลังสอง หรือเรียกว่า Root Mean Square Error (RMSE) ดังนั้นปัจจัยที่ส่งผลต่อ Gait Profile Score (GPS) ได้แก่ ความเอียงตัวและการหมุนของเชิงกราน ความสามารถในการงอ/ เหยียด/หมุน/หุบ/กางข้อสะโพก ความสามารถในการงอ/เหยียดเข่า ความสามารถในการ กระดก/เหยียดข้อเท้า เป็นต้น โดย Gait Profile Score (GPS) ที่มากจะแสดงถึงความผิดปกติที่มาก^17^

หากมีการประมวลผลที่แม่นยำ สร้างระเบียบวิจัยที่ดี กำหนดระยะเวลาการทดลองและกลุ่มตัวอย่างได้ชัดเจน จะนำไปสู่การบอกผลของการนวดต่อการเกร็งของกล้ามเนื้อที่ส่งผลต่อการเดินของผู้ป่วยสมองพิการได้แม่นยำมาก ยิ่งขึ้น แม้ว่าผลลัพธ์จะไม่แตกต่างระหว่างการนวดและการทำกายภาพบำบัด แต่หากมีความแตกต่างภายในกลุ่ม ทดลองอย่างมีนัยสำคัญ การนวดแผนไทยก็อาจเป็นอีกตัวเลือกหนึ่งของการรักษาแบบไม่ใช้การผ่าตัดได้เช่นกัน และถือเป็นการสนับสนุนภูมิปัญญาวิถีไทยในการดูแลรักษาโรค

# วัตถุประสงค์ของโครงการวิจัย

เพื่อศึกษาเปรียบเทียบผลของการนวดแผนไทยโบราณกับการทำกายภาพบำบัดแบบปกติ ในการพัฒนาการเดิน ในผู้ป่วยสมองพิการชนิดกล้ามเนื้อเกร็ง โดยประเมินจากเครื่องวิเคราะห์การเดินออกมาเป็นคะแนน (Gait profile score)

- Primary outcome: Gait profile score (GPS)
- Secondary outcome: Modified Ashworth Scale, Temporal spatial data, Kinetics data, Oxygen consumption, Electromyography และ Pedobarography

# วิธีวิจัยและแบบแผนการวิจัย ตารางการทำวิจัย

รูปแบบการวิจัย : Crossover randomized controlled trial

- ผู้ป่วยที่ตรงตามเกณฑ์และยินยอมเข้าร่วมในการวิจัยจะได้รับการสัมภาษณ์และตรวจร่างกายเพื่อเก็บข้อมูล พื้นฐาน รวมทั้งนัดหมายในการวิเคราะห์การเดิน

- ผู้เข้าร่วมงานวิจัยจะได้รับการวิเคราะห์การเดินโดยเครื่อง Motion capture จากบริษัท Motion analysis corporation, Santa Rosa, CA, USA และบันทึกข้อมูลโดยใช้โปรแกรม Cortex 6.2 วิเคราะห์ข้อมูลโดยใช้ โปรแกรม Orthotrak จากนั้นจะผู้ร่วมงานวิจัยจะถูกสุ่มเป็นกลุ่ม A และ B

- ผู้เข้าร่วมงานวิจัยจะได้รับการสอนการทำกายภาพบำบัดด้วยตนเอง (home program physical therapy) และ ให้ทำตั้งแต่เริ่มงานวิจัยจนจบงานงานวิจัยเป็นเวลา 18 สัปดาห์

- กลุ่ม A จะได้รับการนวดแผนไทยโบราณตามระเบียบที่ได้กล่าวไปข้างต้น เป็นเวลา 6 สัปดาห์ จากนั้นจะ ได้รับการวิเคราะห์การเดินเป็นครั้งที่สอง

- กลุ่ม B จะได้รับการการทำกายภาพบำบัดตามปกติ เป็นเวลา 6 สัปดาห์ จากนั้นจะได้รับการวิเคราะห์การ เดินเป็นครั้งที่สอง

- เมื่อกลุ่ม A หรือ B ได้รับการวิเคราะห์การเดินเป็นครั้งที่สองแล้ว จะให้เว้นช่วงระยะเวลา 6 สัปดาห์ เมื่อ ครบระยะเวลาแล้ว จะได้รับการวิเคราะห์การเดินเป็นครั้งที่สาม

- เมื่อรับการวิเคราะห์การเดินเป็นครั้งที่สามแล้ว จะมีการสลับการรักษาโดยที่กลุ่ม A จะได้รับการทำ กายภาพบำบัดตามปกติ ในขณะที่กลุ่ม B จะได้รับการนวดแผนไทยโบราณ เป็นเวลา 6 สัปดาห์

- จากนั้นทั้งสองกลุ่มจะได้รับการวิเคราะห์การเดินเป็นครั้งที่สี่

- หากมีผู้เข้าร่วมวิจัยไม่สะดวกมาทำกายภาพบำบัดหรือการนวดแผนไทยที่โรงพยาบาลรามาธิบดีทุกสัปดาห์ เนื่องจากอาศัยอยู่ต่างจังหวัด หรือกังวลเรื่องสถานการณ์โรคติดต่อ เช่น โควิด-19 ฯลฯ ผู้เข้าร่วมวิจัย สามารถนำแผนการรักษาของงานวิจัยที่ทำในโรงพยาบาลรามาธิบดี ไปใช้ได้ที่โรงพยาบาลใกล้บ้าน หรือ สามารถกระทำเองที่บ้านได้ เพื่อเป็นการอำนวยความสะดวกแก่ผู้เข้าร่วมวิจัยในสถานการณ์ปัจจุบัน และ ป้องกันการไม่มาติดตามการรักษาในอนาคต (loss to follow up) เนื่องจากเป็นงานวิจัยที่ใช้เวลานาน โดย คณะผู้วิจัยจะทำการสอนขั้นตอนของการทำกายภาพบำบัด และการนวดแผนไทยแก่ผู้ดูแลผู้เข้าร่วม งานวิจัยโดยละเอียด จัดทำวิดิโอสาธิตวิธีการนวดอย่างละเอียด และขั้นตอนเป็นลายลักษณ์อักษร เพื่อ มั่นใจว่าจะสามารถทำเองที่บ้านได้ นอกจากนี้ทางผู้วิจัยจะติดต่อผู้เข้าร่วมวิจัยทุกสัปดาห์เพื่อสอบถาม ปัญหาที่พบ, ตรวจสอบความถูกต้อง และยืนยันว่าได้ทำจริง(Telemedicine) นอกจากนี้ได้มีงานวิจัยของ Paul M Robinson และคณะ^22^ ได้เปรียบเทียบการทำกายภาพบำบัดโดยผู้เชี่ยวชาญ กับทำกายภาพบำบัดเอง ที่บ้าน ในผู้ป่วยไหล่ติดหลังจากได้ฉีดน้ำเข้าข้อไหล่เพื่อทำลายผังผืดแล้ว พบว่า Oxford Shoulder Score(OSS) และ EQ-5D ไม่แตกต่างกันอย่างมีนัยสำคัญ


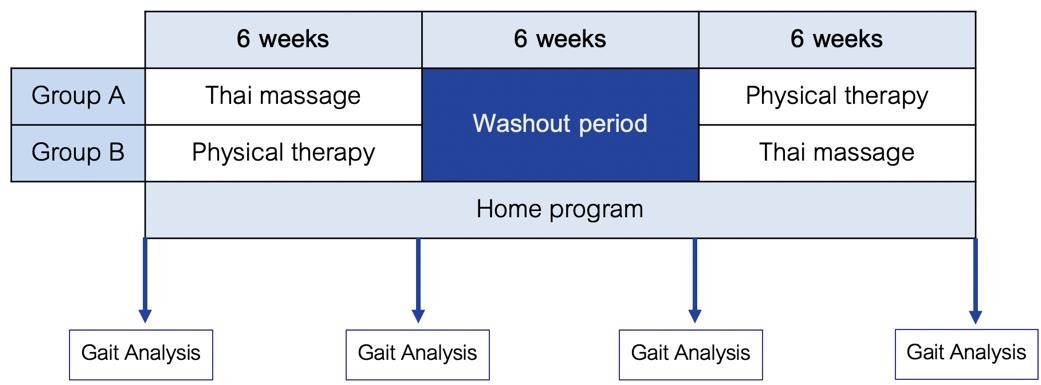


**รูปที่ 3** แผนภูมิการดำเนินการวิจัย

# การนวดแผนไทยโบราณ

ให้การรักษาโดยแพทย์แผนไทยประยุกต์ผู้ที่มีประสบการณ์อย่างน้อย 3 ปี (มีใบประกอบโรคศิลป์) จำนวน 2 คน จะทำการตรวจวินิจฉัยเพื่อประเมินทั้งสภาพร่างกายและสภาพอารมณ์ก่อนเริ่มรักษา การนวดจะใช้

นิ้วหัวแม่มือวางบนตำแหน่งที่ต้องการและกดนวด โดยปรับขนาดและทิศทางของแรงที่ใช้ในการลงน้ำหนักให้ เหมาะสมตามความรุนแรงโรคและตามอายุ อาจพิจารณายืดคลายกล้ามเนื้อด้วยอุ้งมือตามอาการแสดงของโรคอย่าง นุ่มนวล ให้ผู้รับการบำบัดรู้สึกสบายที่สุดและจะไม่กดในลักษณะบด ขยี้ หรือบิดกล้ามเนื้อ

การนวดพื้นฐานจะใช้เวลาจุดละประมาณ 10 วินาที ส่วนการนวดจุดสัญญาณจะใช้เวลาจุดละประมาณ 30 วินาที โดยจะจัดให้ผู้รับการบำบัดอยู่ในท่านอนหงาย นอนตะแคงและท่านั่งตามความเหมาะสม

แพทย์แผนไทยประยุกต์ผู้นวดบำบัดจะดำเนินการรักษาตามมาตรฐานและขั้นตอนที่กำหนด ทั้งนี้วิธีการ นวดบำบัดสามารถปรับตามสถานการณ์โดยขึ้นอยู่กับผู้เข้าร่วมวิจัยเป็นหลัก แต่จะสอดคล้องและเป็นไปตาม ระเบียบปฏิบัติในการนวดไทยโบราณซึ่งมีความปลอดภัย โดยได้พัฒนาแนวทางการกดนวดตามมาตรฐานวิชาการมี ทั้งหมด 10 ขั้นตอน โดยระเบียบและลำดับการนวดแผนไทยโบราณที่ใช้ในงานวิจัย มีดังต่อไปนี้

| 1. นวดแนวเส้นพื้นฐานของขา โดยเริ่มจากน่องขึ้นไปยังต้นขา แล้วย้อนกลับมาที่น่องอีกรอบ จากนั้นกดจุดสัญญาณที่ข้อเท้าด้านหน้าแล้วจึงกดเปิดประตูลมบริเวณขาหนีบ | 5 นาที |
| --- | --- |
| 2. นวดแนวเส้นพื้นฐานของหลังตามแนวกล้ามเนื้อจากระดับ L5 ถึง C7 | 5 นาที |
| 3. นวดจุดสัญญาณของขาด้านนอก โดยเริ่มจากบริเวณสะโพก ไล่ไปตามต้นขาถึงน่องเหนือ ตาตุ่มด้านนอก | 5 นาที |
| 4. นวดจุดสัญญาณของขาด้านใน โดยเริ่มจากบริเวณต้นขาถึงน่องด้านใน | 5 นาที |
| 5. นวดแนวเส้นพื้นฐานแขนด้านใน โดยเริ่มจากกึ่งกลางของต้นแขนด้านในถึงข้อมือด้านหน้า | 2 นาที |
| 6. นวดแนวเส้นพื้นฐานแขนด้านนอกโดยเริ่มจากกึ่งกลางของต้นแขนด้านนอกถึงกึ่งกลางของ ปลายแขนด้านนอก | 2 นาที |
| 7. นวดแนวเส้นพื้นฐานบ่า โดยเริ่มจากขอบบนของสะบักด้านนอกถึงกล้ามเนื้อบริเวณต้นคอ ระดับ C7 | 2 นาที |
| 8. นวดแนวเส้นพื้นฐานเส้นโค้งคอ โดยเริ่มจากจุดบริเวณต้นคอ ระดับ C7 ไล่ไปตามก้านคอถึง บริเวณฐานกะโหลกศีรษะ | 1 นาที |
| 9. นวดจุดสัญญาณ 1, 2 และ 5 ศีรษะด้านหลัง บริเวณฐานกะโหลกศีรษะและนวดจุดจอม ประสาทบริเวณใจกลางกระหม่อมหน้า | 2 นาที |
| 10. นวดจุดสัญญาณ 5 ศีรษะด้านหน้าบริเวณเหนือคาง และจุดเหนือริมฝี ปากบน | 1 นาที |

โดยจะดำเนินการนวดตามระบบที่กล่าวไปเป็นเวลา 2 วัน ต่อสัปดาห์ ครั้งละ 30 นาที ติดต่อกัน 6 สัปดาห์

# Routine Physical Therapy

การทำกายภาพบำบัดเป็นที่นิยมในการรักษาผู้ป่วยสมองพิการ โดยมีจุดประสงค์เพื่อที่จะทำให้พิสัยข้อ (range of motion) และกำลังของกล้ามเนื้อ (muscle strength) ให้คงสภาพเดิม หรือพัฒนาให้ดียิ่งขึ้น นอกจากนี้การ ทำกายภาพบำบัดยังสามารถช่วยพัฒนาการเดินได้อีกด้วย

การทำกายภาพบำบัดในงานวิจัยนี้ทำโดยนักกายภาพบำบัดที่มีประสบการณ์ 5 ปี จำนวน 1 คน (มีใบ ประกอบโรคศิลป์) จะใช้เวลาทั้งหมด 6 สัปดาห์ สัปดาห์ละ 3 ครั้ง ครั้งละ 40 นาที รวม 120 นาที/สัปดาห์ ในแต่ละ ครั้งประกอบด้วย stretching exercise 10 นาที strengthening exercise 15 นาที และ ambulatory training 15 นาที โดย stretching exercise และ strength training จะเลือกทำเฉพาะส่วนของกล้ามเนื้อที่มีปัญหาในแต่ละบุคคล และมีความ ปลอดภัย เช่น gastrosoleus complex, tibialis posterior, peroneal muscle, rectus femoris, hamstring, hip adductor muscle, iliopsoas muscle, biceps brachaii เป็นต้น

# Home Program Physical Therapy

ผู้เข้าร่วมงานวิจัยหรือผู้ปกครองจะได้รับการสอนการทำกายภาพบำบัดเองที่บ้าน โดยกำหนดให้ทำ 5 ครั้ง/ สัปดาห์ ครั้งละอย่างน้อย 30 นาที รวม 150 นาที/สัปดาห์ ประกอบด้วยการทำ stretching exercise โดยยืดข้อที่มี ปัญหาทั้งรยางค์บนและล่าง โดยให้ยืดกล้ามเนื้อจุดละ 30 วินาที จำนวน 3 ครั้งต่อจุด, strengthening exercise โดยให้ ผู้ป่วยทำ shoulder abduction/flexion, elbow flexion/extension, hip extension/ abduction และ knee extension/ flexion ทำซ้˚ากันหลาย ๆ ครั้ง (repetitive movement) ข้อละ 3-5 นาที ทั้งสองข้าง และ ambulatory training เป็นการ ฝึกขยับทั้งแขนและขา ให้เดินหน้า (forward), ถอยหลัง (backward), ซ้ายและขวา (side-to-side) โดยสามารถใช้ เคร่ืองช่วยเดินได้(ถ้ามี)

# Washout Period

งานวิจัยนี้กำหนดให้ washout period มีระยะเวลา 6 สัปดาห์ อ้างอิงจากงานวิจัยของ Esin-Yi Kathy Cheng et al.^20^ ที่มีการทำ cross over randomized controlled trial โดยใช้ washout period 6 สัปดาห์ในกลุ่มเด็กสมองพิการที่ ได้รับการทำการรักษาโดยวิธี whole body vibration เทียบกับการทำกายภาพบำบัด และได้อนุมานจากวรรณกรรมที่ ศึกษาเกี่ยวกับภาวะการเกร็งของกล้ามเนื้อหลังจากมีภาวะหลอดเลือดสมองอุดตัน พบว่าผู้ป่วยเริ่มมีการแข็งเกร็ง ของกล้ามเนื้อที่ 6 สัปดาห์^21^

ใช้เวลา 6 สัปดาห์ โดยระหว่างช่วง washout period ผู้ป่วยยังสามารถใช้อุปกรณ์ช่วยในการเดิน/เฝือก พลาสติก หรือทานยาประจำตัวได้ตามปกติ เพียงแต่ไม่สามารถรับการรักษาอื่นเพิ่มเติมได้ (co-intervention) เช่น ฉีด botulinum A toxin กายภาพบำบัดหรือการนวดใด ๆ เพิ่มเติม ผ่าตัดเพิ่มเติมเพื่อแก้ไขอาการเกร็งตัวกล้ามเนื้อ เป็นต้น และจะมีการโทรศัพท์ติดตามสอบถามอาการ และได้เข้ารับการรักษาอื่นเพิ่มเติมหรือไม่ ทุก 2 สัปดาห์ ในกรณีที่ผู้เข้าร่วมวิจัยมีอุณหภูมิมากกว่า 38.0 องศาเซลเซียส จะไม่สามารถเข้ารับการทำภาพบำบัดได้ รวมถึงการ นวดแผนไทยเช่นกัน โดยผู้ป่วยสามารถเข้ารับการรักษาได้ในอีก 1 สัปดาห์ถัดไป หรือเร็วกว่านั้น แต่ถ้าหากผู้ป่วย

ยังมีไข้เกิน 1 สัปดาห์ และยังไม่สามารถหาสาเหตุได้ หรือเป็นไข้ที่ต้องใช้เวลาการรักษายาวนาน เช่น มะเร็งเม็ด เลือดขาว ไข้มาลาเรีย วัณโรคปอด ฯลฯ จะมีความจำเป็นที่จะต้องถอนตัวจากงานวิจัย แต่ข้อมูลผู้ป่วยเข้าร่วมวิจัยยัง จะคงนำมาคิดวิเคราะห์เช่นเดิม

เนื่องจาก Home program จะเริ่มทำตั้งแต่หลังจากเริ่มวิเคราะห์การเดินครั้งที่ 1 จนจบการทดลอง โดย ผู้เข้าร่วมวิจัยเป็นผู้ทำเอง หรือผู้ปกครองเป็นผู้ช่วยในการทำ ดังนั้นในช่วง washout period ก็จะให้ผู้เข้าร่วมวิจัยทำ Home program เหมือนช่วงที่ได้รับการรักษาโดยการนวดแผนไทย หรือการทำกายภาพบำบัดตามวิธีที่ได้ระบุไว้ ข้างต้น

# Gait analysis

การวิเคราะห์การเดินมีขั้นตอนดังนี้

1. สวมชุดที่สะดวกต่อการติดเครื่องมือวัดการเคลื่อนไหวและคลื่นไฟฟ้ากล้ามเนื้อบนผิวหนัง

2. ทำการติดอุปกรณ์จับการเคลื่อนไหวทั้งหมด 29 ตำแหน่งตามวิธีการของ Helen Hayes

1. ทำการติดอุปกรณ์วัดคลื่นไฟฟ้ากล้ามเนื้อโดยใช้ bipolar Ag/AgCl electrodes (3M Red dot, size 35 × 40 cm) ที่ ผิวหนังตามตำแหน่งต่างๆ ได้แก่ กล้ามเนื้อสะโพก, กล้ามเนื้อเรกตัส ฟีเมอริส, กล้ามเนื้อแฮมสตริง, กล้ามเนื้อ หน้าแข้งทางด้านหน้า และกล้ามเนื้อส่วนน่องทางด้านหลัง
2. สวมอุปกรณ์เพื่อทำการวัด oxygen consumption โดยใช้เคร่ืองมือ Oxycon mobile โดยบริษัท CareFusion จาก ประเทศเยอรมนี
3. วัดแรงของกล้ามเนื้อที่มากที่สุดที่สามารถทำได้ของกล้ามเนื้อแต่ละกลุ่มตามที่ได้กล่าวไว้ข้างต้น เพื่อใช้เป็น ข้อมูลพื้นฐานในการวิเคราะห์แต่ละคน โดยใช้ ProEMG software, Myon 320 wireless EMG (Myon AG, Schwarzenberg Switzerland)
4. ให้ผู้เข้าร่วมวิจัยเดินเป็นแนวเส้นตรงระยะประมาณ 8 เมตร จำนวณ 10-15 รอบ ด้วย gait pattern เดิม โดยจะมี กล้องจับการเคลื่อนไหวของข้อและตัววัดแรงกระทำต่อพื้น วัดโดย 3D motion capture and Coretex 6.2 software วิเคราะห์ข้อมูลด้วย OrthoTRack 6.61 (Motion AnalysisCorporation, Santa Rosa, CA, USA) และวัด คลื่นไฟฟ้ากล้ามเนื้อขณะเดิน เลือก 3 รอบที่ดีที่สุดเพื่อนำมาใช้ในการคำนวณจลศาสตร์และจลนพลศาสตร์ของ ข้อ (ในกรณีที่ไม่สามารถติดอุปกรณ์ทั้งสองอย่างพร้อมกันได้ จะทำการติดและวัดทีละอย่าง)
5. ให้ผู้เข้าร่วมวิจัยยืนและเดินบนเครื่องวัดการลงน้ำหนักเท้าเพื่อวัดการลงน้ำหนักเท้าขณะยืนและเดินโดยใช้ เครื่อง Sensor Medica Pedobarograph (Rome, Italy)


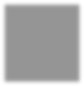

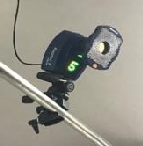

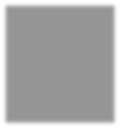

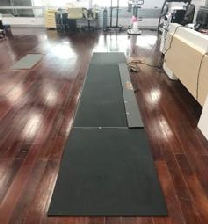

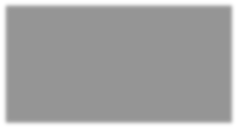

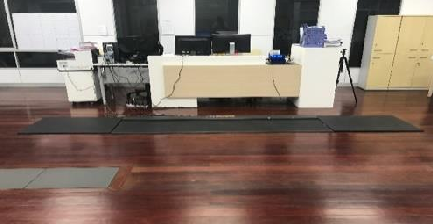

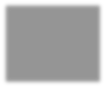

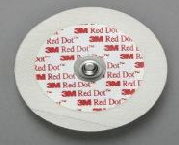

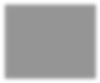

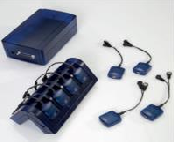


**รูปที่ 3** อุปกรณ์การตรวจ 3D gait analysis

ตารางการทำวิจัย

|  | ก.ค. 64 – ก.ค. 65 | ก.ค. 65 – ก.พ. 66 | ก.พ. 66 – พ.ค. 66 |
| --- | --- | --- | --- |
| Subject recruitment & operation |  |  |  |
| Data collection |  |  |  |
| Data analysis |  |  |  |

# Protocol Flow Chart

**
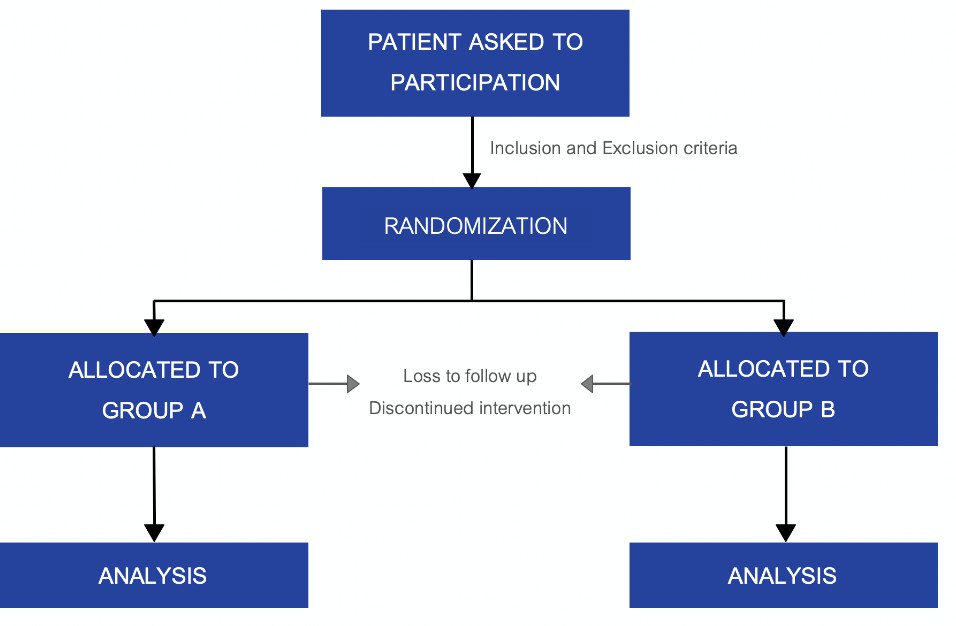
**

**รูปที่ 4** แผนภูมิการดำเนินการวิจัย CONSORT diagram

# ระบุจำนวนผู้เข้าร่วมการวิจัย (Subject) ที่จะศึกษา พร้อมทั้งเกณฑ์คัดเข้าและเกณฑ์คัดออก

อ้างอิงจากค่าเฉลี่ย GPS (Rasmussen HM, Nielsen DB, Pedersen NW, Overgaard S, Holsgaard-Larsen A. Gait Deviation Index, Gait Profile Score and Gait Variable Score in children with spastic cerebral palsy: Intra-rater reliability and agreement across two repeated sessions. Gait Posture. 2015;42(2):133-7)

ใช้การคำนวณด้วยโปรแกรม STATA 16.0, Stata Corp, College Station, Texas, USA เพื่อประเมินขนาด ตัวอย่างชนิด difference two independent mean test เนื่องจากเป็นการศึกษาชนิด randomized controlled trial โดย กำหนดค่าตัวแปรดังนี้

- กำหนด Alpha error เท่ากับ 0.05 และ power of the study เท่ากับ 0.8

- ค่าเฉลี่ยของ Gait Profile Score (GPS) ของผู้ป่วย spastic cerebral palsy กลุ่มควบคุม มีค่า 13.90

- ค่าความแตกต่างของค่าเฉลี่ยของ Gait Profile Score (GPS) ระหว่างกลุ่มทดลอง กับกลุ่มควบคุมสำหรับ alternate hypothesis มีค่าร้อยละ 20 หรือคิดเป็น 11.12

- ส่วนเบี่ยงเบนมาตรฐานของ Gait Profile Score (GPS) ของกลุ่มควบคุม มีค่า 4.9

- ส่วนเบี่ยงเบนมาตรฐานของ Gait Profile Score (GPS) ของกลุ่มทดลอง มีค่า 1.3

คำนวณขนาดตัวอย่างได้กลุ่มละ 26 คน

# เผื่อขนาดตัวอย่างเพิ่มขึ้นอีกร้อยละ 20 ได้ขนาดตัวอย่างทั้งสิ้นกลุ่มละ 32 คน

**เกณฑ์การคัดเลือกอาสาสมัครเข้าโครงการวิจัย ( Inclusion Criteria )**

1. ผู้ป่วยสมองพิการชนิดกล้ามเนื้อเกร็ง (spastic cerebral palsy)
2. อายุมากกว่า 5 ปี
3. Gross Motor Function Classification System (GMFCS) level I ถึง III (รูปที่ 2 และ 3)
4. ยินยอมเข้าร่วมโครงการวิจัยโดยการลงนาม

# เกณฑ์การคัดเลือกอาสาสมัครออกจากโครงการวิจัย (Exclusion Criteria)

1. อุณหภูมิร่างกายมากกว่า 38.0 องศาเซลเซียส
2. มีภาวะเลือดออกง่ายหรือหยุดยาก
3. มีภาวะกระดูกหักที่ยังไม่ติด
4. มีภาวะข้อติด (fixed joint contracture)
5. มีภาวะผิวหนังติดเชื้อ
6. มีผื่นแพ้ผิวหนัง (contact dermatitis)
7. ผู้ป่วยที่ได้รับการรักษาโดยการฉีด Botulinum A toxin ภายใน 6 เดือน
8. มีภาวะสมาธิสั้น (attention deficit hyperactive disorder)
9. มีภาวะพฤติกรรมผิดปกติ (behavioral disorder)
10. มีโรคลมชักที่ควบคุมไม่ได้ (uncontrolled epilepsy)
11. ผู้ป่วยปฏิเสธหรือขอถอนตัวออกจากโครงการวิจัย

# ระยะเวลาในการศึกษา (ต้องเริ่มหลังจากได้รับการอนุมัติจากคณะกรรมการจริยธรรมการวิจัยในคนแล้ว)

หลังจากได้รับอนุมัติจริยธรรมการวิจัยในคน เป็นระยะเวลา 22 เดือน

# ความเสี่ยงหรือความไม่สบายที่คาดว่าจะเกิดขึ้นกับผู้เข้าร่วมการวิจัย

การเกิดการบาดเจ็บระหว่างการนวดแผนไทยโบราณและการทำกายภาพบำบัด เช่น การมีกล้ามเนื้ออักเสบ รอยฟกช้ำจากการนวด หรือแม้แต่อุบัติเหตุจากการเดินขณะเข้าร่วมการวิเคราะห์การเดิน เช่นการหกล้ม

ทางคณะผู้วิจัยมีมาตรการป้องกันการเกิดกล้ามเนื้ออักเสบหรือรอยฟกช้ำจากการนวด โดยจัดสรร แพทย์แผนไทยประยุกต์และนักกายภาพบำบัดผู้ที่มีใบประกอบโรคศิลป์ และมีประสบการณ์ในการทำงาน เป็นผู้ให้การรักษาแก่ผู้เข้าร่วมวิจัย และจะมีการประเมินความตึงของกล้ามเนื้อ และให้การรักษาที่เหมาะสมแก่ ผู้เข้าร่วมวิจัยแต่ละราย ซึ่งหากเกิดเหตุการณ์ไม่พึงประสงค์ข้างต้น ผู้วิจัยจะให้การดูแลรักษาตามมาตรฐานทาง การแพทย์อย่างสุดความสามารถภายใต้สิทธิการรักษาของผู้เข้าร่วมวิจัย

ในส่วนของการเกิดอุบัติเหตุขณะวิเคราะห์การเดิน คณะผู้วิจัยจะทำการซักประวัติ ตรวจร่างกายทุกครั้งเพื่อ ประเมินว่าผู้เข้าร่วมวิจัยสามารถเดินติดต่อกันตามที่กำหนดได้หรือไม่ โดยหากผู้เข้าร่วมวิจัยต้องใช้เครื่องช่วย เดินอยู่แล้วในชีวิตประจำวัน ก็จะสามารถใช้ขณะวิเคราะห์การเดินได้เช่นเดิม นอกจากนี้ ก่อนการวิเคราะห์การ เดินทุกครั้ง จะมีการตรวจสอบสภาพแวดล้อมในห้องว่ามีความเสี่ยงในการเกิดอุบัติเหตุหรือไม่ เช่น ความลื่น ของพื้น สิ่งของที่กีดขวางการเดิน แสงไฟในห้อง เป็นต้น โดยหากเกิดเหตุการณ์ไม่พึงประสงค์ข้างต้น ผู้วิจัย จะให้การดูแลรักษาตามมาตรฐานทางการแพทย์อย่างสุดความสามารถภายใต้สิทธิการรักษาของผู้เข้าร่วมวิจัย

1. **ประโยชน์ที่คาดว่าจะได้รับ** ได้รับข้อมูลเกี่ยวกับการเดินจากเครื่องวิเคราะห์การเดินอย่างละเอียดเพื่อที่จะแก้ปัญหาเกี่ยวกับการเดินได้ตรง จุดมากยิ่งขึ้น

# ข้อพิจารณาด้านจริยธรรม

ผู้เข้าร่วมการทดลองจะได้รับการอธิบายถึงแนวทางการดำเนินงานวิจัยทุกขั้นตอน ประโยชน์และโทษที่อาจ จะเกิดขึ้นต่อผู้ป่วยอย่างละเอียด

# ค่าชดเชยแก่ผู้เข้าร่วมการวิจัย (ตามความจำเป็นและเหมาะสม) ในกรณีที่เกิดอันตรายหรือผลอันไม่พึง ประสงค์ ต่อผู้เข้าร่วมการวิจัยนี้ อาสาสมัครจะได้รับการดูแลรักษาโดยไม่ต้องเสียค่าใช้จ่ายอย่างใดบ้าง

ผู้เข้าร่วมการวิจัยจะได้รับเงินค่าตอบแทนจำนวน 3,000 บาทตลอดโครงการ ทางผู้วิจัยจะรับผิดชอบดูแลและ ป้องกันมิให้เกิดภาวะแทรกซ้อนจากการวิจัย หากเกิดภาวะดังกล่าว ผู้วิจัยจะให้การดูแลรักษาตามมาตรฐานทาง การแพทย์อย่างสุดความสามารถภายใต้สิทธิการรักษาของผู้เข้าร่วมวิจัย ผู้เข้าร่วมการวิจัยจะได้รับการรักษาตาม มาตรฐานโดยไม่เสียค่าใช้จ่ายจนกว่าผลเสียจากการวิจัยจะทุเลาลง

# ระบุแหล่งทุนสนับสนุนในกรณีที่ได้รับทุนสนับสนุนจากภาคเอกชนให้แจกแจงรายละเอียดของงบประมาณ และใส่ชื่อผู้ประสานงานของผู้ให้ทุน พร้อมเบอร์โทรศัพท์ที่สามารถติดต่อได้

อยู่ในระหว่างดำเนินการขอทุนวิจัยจากคณะแพทยศาสตร์โรงพยาบาลรามาธิบดี

# เอกสารชี้แจงข้อมูลและคำแนะนำแก่ผู้เข้าร่วมโครงการ(Patient/Participant Information Sheet)

- ตามเอกสารแนบ –

# หนังสือยินยอมโดยได้รับการบอกกล่าวและเต็มใจ (Informed Consent Form)

- ตามเอกสารแนบ –

# หลักฐานหรือข้อมูลอ้างอิง

1. Graham HK, Thomason P, Novacheck TF. Cerebral Palsy. In: Stuart L. Weinstein M, John M. (Jack) Flynn M, editors. Lovell and Winter’s pediatric orthopedics. 1. 7th ed. Philadelphia2014. p. 484-549.
2. MacLennan AH, Thompson SC, Gecz J. Cerebral palsy: causes, pathways, and the role of genetic variants. Am J Obstet Gynecol. 2015;213(6):779-88.
3. Korzeniewski SJ, Slaughter J, Lenski M, Haak P, Paneth N. The complex aetiology of cerebral palsy. Nat Rev Neurol. 2018;14(9):528-43.
4. Bax M, Goldstein M, Rosenbaum P, Leviton A, Paneth N, Dan B, et al. Proposed definition and classification of cerebral palsy, April 2005. Dev Med Child Neurol. 2005;47(8):571-6.
5. Colver A, Fairhurst C, Pharoah PO. Cerebral palsy. Lancet. 2014;383(9924):1240-9.
6. Franki I, Desloovere K, De Cat J, Feys H, Molenaers G, Calders P, et al. The evidence-base for basic physical therapy techniques targeting lower limb function in children with cerebral palsy: a systematic review using the International Classification of Functioning, Disability and Health as a conceptual framework. J Rehabil Med. 2012;44(5):385-95.
7. Thanakiatpinyo T, Suwannatrai S, Suwannatrai U, Khumkaew P, Wiwattamongkol D, Vannabhum M, et al. The efficacy of traditional Thai massage in decreasing spasticity in elderly stroke patients. Clin Interv Aging. 2014;9:1311-9.
8. วรชาติ เฉิดชมจันทร์. คณบดีกายภาพบำบัดห่วง ‘นักกายภาพบำบัดชุมชน’ ภาครัฐขาดแคลน เหตุระบบไม่ จูงใจ 2018 [cited 2020. Available from: [www.hfocus.org/content/2018/01/15261.](http://www.hfocus.org/content/2018/01/15261)
9. Negahban H, Rezaie S, Goharpey S. Massage therapy and exercise therapy in patients with multiple sclerosis: a randomized controlled pilot study. Clin Rehabil. 2013;27(12):1126-36.
10. Cambron JA, Dexheimer J, Coe P, Swenson R. Side Effects of Massage Therapy; a Pilot Study. National University of Health Sciences.
11. Alizad V, Vameghi R, Sajedi F, Alaeddini F, Jazy MRH. Swedish massage and abnormal reflexes of children with spastic cerebral palsy. Iranian Rehabilitation Journal. 2007;5(5,6):30-3.
12. Rasool F, Memon AR, Kiyani MM, Sajjad AG. The effect of deep cross friction massage on spasticity of children with cerebral palsy: A double-blind randomised controlled trial. J Pak Med Assoc. 2017;67(1):87-91.
13. Malila P, Seeda K, Machom S, Eungpinithpong W. Effects of Thai Massage on Spasticity in Young People with Cerebral Palsy. J Med Assoc Thai. 2015;98 Suppl 5:S92-6.
14. Mahmood Q, Habibullah S, Babur MN. Potential effects of traditional massage on spasticity and gross motor function in children with spastic cerebral palsy: A randomized controlled trial. Pak J Med Sci. 2019;35(5):1210-5.
15. Hernandez-Reif M, Feild T, Largie S, Diego M, Manigat N, Seoanes J, et al. Cerebral palsy symptoms in children decreased following massage therapy. Early Child development and Care. 2005;175(5):445-56.
16. Armand S, Decoulon G, Bonnefoy-Mazure A. Gait analysis in children with cerebral palsy. EFORT open reviews. 2016;1:448-58.
17. Holmes SJ, Mudge AJ, Wojciechowski EA, Axt MW, Burns J. Impact of multilevel joint contractures of the hips, knees and ankles on the Gait Profile score in children with cerebral palsy. Clin Biomech (Bristol, Avon). 2018;59:8-14.
18. Baker R, McGinley JL, Schwartz MH, Beynon S, Rozumalski A, Graham HK, et al. The gait profile score and movement analysis profile. Gait Posture. 2009;30(3):265-9.
19. อภิชาติ ลิมติยะโยธิน, สมพร หนองบัวดี. บทนำการนวดไทยแบบราชสำนัก. In: โรงเรียนอายุรเวทธ˚ารง สถานการแพทย์แผนไทยประยุกต์, คณะแพทย์ศาสตร์ศิริราชพยาบาล มหาวิทยาลัยมหิดล, editors. หัตถเวช กรรมแผนไทย(การนวดไทยแบบราชสำนัก) ตอนที่ 1: การนวดพื้นฐาน. 1. 2 ed2557. p. 1-8.
20. Cheng HY, Yu YC, Wong AM, Tsai YS, Ju YY. Effects of an eight-week whole body vibration on lower extremity muscle tone and function in children with cerebral palsy. Res Dev Disabil. 2015;38:256-61.
21. Thibaut A, Chatelle C, Ziegler E, Bruno MA, Laureys S, Gosseries O. Spasticity after stroke: physiology, assessment and treatment. Brain Inj. 2013;27(10):
22. Robinson PM, Norris J, Roberts CP. Randomized controlled trial of supervised physiotherapy versus a home exercise program after hydrodilatation for the management of primary frozen shoulder. J Shoulder Elbow Surg. 2017 May;26(5):757-765. doi: 10.1016/j.jse.2017.01.012. Epub 2017 Mar 18. PMID: 28318848.

**ภาคผนวก**

**แบบบันทึกข้อมูล**

| **ส่วนที่ 1** : ข้อมูลผู้เข้าร่วมวิจัยเบื้องต้น | | สำหรับเจ้าหน้าที่กรอกเท่านั้น |
| --- | --- | --- |
| 1.1  1.2  1.3  1.4  1.5  1.6  1.7  1.8  1.9  1.10  1.11  1.12  1.13 | วันที่เข้าร่วมงานวิจัย: ………/………../………  ชื่อ-สกุล: ……………..…………………………………………………...  เพศ ❒ 1.ชาย ❒ 2. หญิง  อายุ: ………. ปี  น้ำหนัก: …………. กิโลกรัม  ส่วนสูง: ………….. เซนติเมตร  รูปแบบการเกร็งของกล้ามเนื้อ:  ❒ 1. spastic diplegia  ❒ 2. spastic hemiplegia  ❒ 3. spastic quadriplegia  Gross Motor Function Classification System   (GMFCS) level  ❒ 1. I ❒ 2. II ❒ 3. III ❒ 4. IV  น้ำหนักแรกคลอด:  ❒ 1. < 1000 กรัม  ❒ 2. 1000 – 1499 กรัม  ❒ 3. 1500 – 2500 กรัม  ❒ 4. > 2500 กรัม  รูปแบบการคลอด: ❒ 1.คลอดธรรมชาติ ❒ 2.ผ่าตัดคลอด  สถานที่เกิด:  ❒ 1.โรงพยาบาลรัฐบาล ❒ 2. โรงพยาบาลเอกชน  ภาวะแทรกซ้อนขณะคลอด/หลังคลอด:  …………………………………………………………………  การรักษาก่อนเข้าร่วมงานวิจัยและปีพ.ศ.:  ………………………………………………………………… | Date [ ][ ] / [ ][ ] / [ ][ ]  Name [ ]  Sex [ ]  Age [ ]  Weight [ ] [ ] [ ]  Height [ ] [ ] [ ]  Spastic type [ ]  GMFCS [ ]  Birth weight [ ]  Mode of delivery [ ]  Birth place [ ]  Complication [ ]  Previous treatment [ ] |

**ส่วนที่ 2:** การตรวจร่างกาย

2.1 Mobility scales

|  | | Passive ROM | | Active ROM | | Muscle Power | |
| --- | --- | --- | --- | --- | --- | --- | --- |
|  |  | Right | Left | Right | Left | Right | Left |
| 2.1.1 | Hip Flexion |  |  |  |  |  |  |
| 2.1.2 | Hip Extension |  |  |  |  |  |  |
| 2.1.3 | Hip Abduction |  |  |  |  |  |  |
| 2.1.4 | Hip Adduction |  |  |  |  |  |  |
| 2.1.5 | Hip Internal Rotation |  |  |  |  |  |  |
| 2.1.6 | Hip External Rotation |  |  |  |  |  |  |
| 2.1.7 | Knee Flexion |  |  |  |  |  |  |
| 2.1.8 | Knee Extension |  |  |  |  |  |  |
| 2.1.8 | Popliteal Angle |  |  | - | - | - | - |
| 2.1.9 | Ely’s test |  |  | - | - | - | - |
| 2.1.10 | Ankle Dorsiflexion with knee extension |  |  |  |  |  |  |
| 2.1.11 | Ankle Dorsiflexion with knee flexion |  |  |  |  |  |  |
| 2.1.12 | Ankle Plantar flexion |  |  |  |  |  |  |
| 2.1.13 | Ankle Inversion |  |  |  |  |  |  |
| 2.1.14 | Ankle Eversion |  |  |  |  |  |  |

2.2 Motor control

| Muscle | | Right | Left | **Good**: Patient is able to isolate individual muscle contraction through entire available passive ROM upon command.  **Fair**: Patient is able to initiate muscle contraction upon command, but it unable to completely isolate contraction through entire passive ROM.  **Poor**: Patient is unable to isolate individual muscle contraction through entire available passive ROM secondary to synergistic patterns, increase tone and/or decrease or absent activation. |
| --- | --- | --- | --- | --- |
| 2.2.1 | Iliopsoas |  |  |  |
| 2.2.2 | Gluteus Maximus |  |  |  |
| 2.2.3 | Quadriceps |  |  |  |
| 2.2.4 | Hamstring |  |  |  |
| 2.2.5 | Anterior Tibialis |  |  |  |
| 2.2.6 | Gastrocnemius |  |  |  |
| 2.2.7 | Posterior Tibialis |  |  |  |
| 2.2.8 | Soleus |  |  |  |
| 2.2.9 | Peroneus |  |  |  |

2.3 Muscle tone

| Muscle | | Right | Left | **Modified Ashworth scale**  0: No increase in tone  1: Slight increase in muscle tone, manifested by a catch and release or minimal resistance at  the end of the ROM when the affected part(s) is moved in flexion or extension  1+: Slight increase in muscle tone, manifested by a catch, followed by minimal resistance  throughout the remainder (less than half) of the ROM  2: More marked increase in muscle tone through most of the ROM, but affected part(s) easily  moved  3: Considerable increase in muscle tone, passive movement difficult  4: Affected part(s) rigid in flexion or extension |
| --- | --- | --- | --- | --- |
| 2.3.1 | Iliopsoas |  |  |  |
| 2.3.2 | Gluteus Maximus |  |  |  |
| 2.3.3 | Adductor |  |  |  |
| 2.3.4 | Rectus Femoris |  |  |  |
| 2.3.5 | Hamstring |  |  |  |
| 2.3.6 | Anterior Tibialis |  |  |  |
| 2.3.7 | Gastrocnemius |  |  |  |
| 2.3.8 | Posterior Tibialis |  |  |  |
| 2.3.9 | Soleus |  |  |  |
| 2.3.10 | Peroneus |  |  |  |
| 2.3.11 | Clonus |  |  |  |

**ส่วนที่ 3:** 3D gait analysis

- 1. Temporal spatial

|  | | | **Subject** | **St.Dev** | **Norm** | **St.Dev** | **%Norm** |
| --- | --- | --- | --- | --- | --- | --- | --- |
| 3.1.1 | Velocity | (cm/s) |  |  |  |  |  |
| 3.1.2 | Cadence | (steps/min) |  |  |  |  |  |
| 3.1.3 | Stride Length | (cm) |  |  |  |  |  |
| 3.1.4 | Step Width | (cm) |  |  |  |  |  |
| 3.1.5 | Pelvic Width | (cm) |  |  |  |  |  |
| 3.1.6 | Pelvic to Step Ratio | (cm) |  |  |  |  |  |
|  |  |  |  |  |  |  |  |
|  |  |  |  |  |  |  |  |
| **Right** | | | **Subject** | **St.Dev** | **Norm** | **St.Dev** | **%Norm** |
| 3.1.7 | Step Length | (cm) |  |  |  |  |  |
| 3.1.8 | Weight Accept | (% cycle) |  |  |  |  |  |
| 3.1.9 | Single Support | (% cycle) |  |  |  |  |  |
| 3.1.10 | Weight Release | (% cycle) |  |  |  |  |  |
| 3.1.11 | Stance | (% cycle) |  |  |  |  |  |
| 3.1.12 | Swing | (% cycle) |  |  |  |  |  |
|  |  |  |  |  |  |  |  |
|  |  |  |  |  |  |  |  |
| **Left** | | | **Subject** | **St.Dev** | **Norm** | **St.Dev** | **%Norm** |
| 3.1.13 | Step Length | (cm) |  |  |  |  |  |
| 3.1.14 | Weight Accept | (% cycle) |  |  |  |  |  |
| 3.1.15 | Single Support | (% cycle) |  |  |  |  |  |
| 3.1.16 | Weight Release | (% cycle) |  |  |  |  |  |
| 3.1.17 | Stance | (% cycle) |  |  |  |  |  |
| 3.1.18 | Swing | (% cycle) |  |  |  |  |  |

- The patient walked ………. cm/sec with cadence at ………. steps/min.
- Stride length was ………. cm. and step width was ………. cm.
- Right step length was ………. cm. and the left was ………. cm.
- Stance/Swing phase was ……….% and ……….% at the right limb; ……….% and ……….% at the left side, respectively

3.2 Kinematic

**Right lower extremity kinematic**

…………………….……………………………………………………………………………………………………………………………………………………………………………………………………………………………………………………………………………………………………………………………………………………………………………………………………………………………………………………………………………………………………………………………………………………………………………………………………………………………………………………………………………………………………………………………………………………………………………………………………………………………………………………………………………………………………………………………………………………………………………………………………………………………………………………………………………………………………………………………………………………………………………………………………………………………………………………………………………………………………………………………………………………………………………………………………………………………………………………………………………………

**Left lower extremity kinematic**

…………………….……………………………………………………………………………………………………………………………………………………………………………………………………………………………………………………………………………………………………………………………………………………………………………………………………………………………………………………………………………………………………………………………………………………………………………………………………………………………………………………………………………………………………………………………………………………………………………………………………………………………………………………………………………………………………………………………………………………………………………………………………………………………………………………………………………………………………………………………………………………………………………………………………………………………………………………………………………………………………………………………………………………………………………………………………………………………………………………………………………………

3.3 Kinetic

**Right kinetic**

…………………….……………………………………………………………………………………………………………………………………………………………………………………………………………………………………………………………………………………………………………………………………………………………………………………………………………………………………………………………………………………………………………………………………………………………………………………………………………………………………………………………………………………………………………………………………………………………………………………………………………………………………………………………………………………………………………………………………………………………………………………………………………………………………………………………………………………………………………………………………………………………………………………………………………………………………………………………………………………………………………………………………………………………………………………………………………………………………………………………………………………

**Left kinetic**

…………………….……………………………………………………………………………………………………………………………………………………………………………………………………………………………………………………………………………………………………………………………………………………………………………………………………………………………………………………………………………………………………………………………………………………………………………………………………………………………………………………………………………………………………………………………………………………………………………………………………………………………………………………………………………………………………………………………………………………………………………………………………………………………………………………………………………………………………………………………………………………………………………………………………………………………………………………………………………………………………………………………………………………………………………………………………………………………………………………………………………………

3.4 Trunk and Pelvis

…………………….…………………………………………………………………………………………………………………………………………………………………………………………………………………………………………………………………………………………………………………………………………………………………………………………………………………………………………………………………………………………………………………………………………

3.5 Muscle activity

…………………….……………………………………………………………………………………………………………………………………………………………………………………………………………………………………………………………………………………………………………………………………………………………………………………………………………………………………………………………………………………………………………………………………………………………………………………………………………………………………………………………………………………………………………………………………………………………………………………………………………………………………………………………………………………………………………………………………………………………………………………………………………………………………………………………………………………………………………………………………………………………………………………………………………………………………………………………………………………………………………………………………………………………………………………………………………………………………………………………………………………

3.6 Oxygen consumption

…………………….……………………………………………………………………………………………………………………………………………………………………………………………………………………………………………………………………………………………………………………………………………………………………………………………………………………………………………………………………………………………………………………………………………………………………………………………………………………………………………………………………………………………………………………………………………………………………………………………………………………………………………………………………………………………………………………………………………………………………………………………………………………………………………………………………………………………………………………………………………………………………………………………………………………………………………………………………………………………………………………………………………………………………………………………………………………………………………………………………………………
